# WHO Critical Priority Fungi: A Comprehensive Review of Antifungal Resistance, Emerging Therapeutic Strategies, and the Potential of Antifungal Peptides

**DOI:** 10.3390/ijms27188141

**Published:** 2026-09-12

**Authors:** Ekaterina I. Finkina, Olga V. Shevchenko, Anastasia A. Gerasimova, Serafima I. Fateeva, Sergey V. Balandin, Tatiana V. Ovchinnikova

**Affiliations:** M.M. Shemyakin & Yu.A. Ovchinnikov Institute of Bioorganic Chemistry, Russian Academy of Sciences, Miklukho-Maklaya Str., 16/10, 117997 Moscow, Russiaovch@ibch.ru (T.V.O.)

**Keywords:** critical priority fungi, *Candida albicans*, *Candida auris*, *Aspergillus fumigatus*, *Cryptococcus neoformans*, resistance, antifungal peptides

## Abstract

Extensive clinical studies have convincingly demonstrated that fungal infections constitute a mounting global health concern. *Candida albicans*, *C. auris*, *Aspergillus fumigatus*, and *Cryptococcus neoformans* were classified by the WHO as critical priority fungi due to their widespread prevalence, high mortality rates associated with invasive mycoses, and the increasing spread of resistant strains. The currently available antifungal arsenal, comprising four major classes (azoles, polyenes, echinocandins, and flucytosine), is critically insufficient. Resistance to these agents is escalating worldwide, while the approval of new drugs remains alarmingly slow. This review provides a comprehensive overview of the molecular mechanisms underlying resistance to conventional and emerging antimycotics in critical priority fungi, including recently approved and investigational agents such as oteseconazole, rezafungin, ibrexafungerp, fosmanogepix, and olorofim. It examines current strategies to overcome resistance, including combination therapy, drug repurposing, targeted delivery, antibiofilm approaches, immunotherapy, and the application of natural antifungal compounds. Special emphasis is placed on antifungal peptides (AFPs) derived from plants, animals, bacteria, and fungi, along with their synthetic analogs, all of which have demonstrated efficacy against critical priority fungi. The review primarily focuses on the molecular targets of AFPs and on the development of fungal resistance under prolonged exposure, compared with conventional antifungals, in order to evaluate their potential as prototypes for next-generation antimycotics.

## 1. Introduction

Fungal diseases range from common skin and mucous membrane infections to severe invasive mycoses associated with high mortality. While long underestimated, global clinical and surveillance data clearly show that fungal infections represent a critical challenge to human health [1].

Superficial mycoses are the most common fungal infections. According to an analysis of the Global Burden of Disease data, approximately 1.73 billion cases of fungal skin diseases occurred worldwide in 2021, and projections indicate a substantial increase in both incidence and prevalence [2]. Although superficial mycoses rarely pose a direct threat to life, they may become chronic or recurrent, require prolonged or repeated treatment, and markedly reduce the quality of life [3]. Invasive mycoses pose the greatest threat because the pathogen enters the bloodstream, internal organs, and deep tissues. Current model-based estimates suggest that approximately 6.5 million invasive fungal infections occur annually and are associated with about 3.8 million deaths [4].

Fungal infections mainly occur when the immune system is weakened. Patients with oncological and hematological diseases, HIV infection, diabetes mellitus, tuberculosis, and chronic lung diseases are especially vulnerable. The risk of mycoses also increases with prolonged stays in intensive care units, the use of invasive devices, transplantation, major surgery, treatment with broad-spectrum antibiotics, and immunosuppressive therapy. Host genetic predisposition and immunological vulnerability also play important roles [5,6,7,8]. The COVID-19 pandemic further exacerbated the problem by increasing the number of critically ill patients with several simultaneous risk factors [9,10,11].

In 2022, the World Health Organization (WHO) published its first fungal priority pathogens list to draw global attention to the growing problem. *Candida albicans*, *C. auris* (reclassified based on genomic taxonomy as *Candidozyma auris*), *Aspergillus fumigatus*, and *Cryptococcus neoformans* were assigned to the critical priority group due to their widespread prevalence, high mortality rates from invasive infections, and the spread of resistant fungal strains [12]. *C. albicans* is responsible for opportunistic infections and is a common cause of systemic mycoses. While invasive candidiasis generally responds to treatment, its high adaptability poses a risk of resistance development [13]. *C. auris* is relatively rare but is characterized by high resistance to disinfectants, persistence on surfaces, and a tendency to cause outbreaks of invasive candidiasis in healthcare facilities. Furthermore, *C. auris* often exhibits multidrug resistance [14]. Among people with HIV, approximately 2 million cases of oral candidiasis and 1.3 million cases of esophageal candidiasis occur each year [15]. Recurrent vulvovaginal candidiasis affects approximately 138 million women worldwide every year [16]. Candidemia and invasive candidiasis account for approximately 1.5 million new cases each year, with 995,000 resulting in death [4]. *Cr. neoformans* is transmitted to humans through the inhalation of spores and causes cryptococcal meningitis, a challenging condition characterized by relapses, a refractory course, and a high mortality rate. Treatment involves antimycotics from several classes; therefore, fungal resistance poses a significant threat to therapeutic efficacy [17,18]. Cryptococcal meningitis is diagnosed in approximately 200,000 people annually, and 75.8% of these cases result in death [4]. *A. fumigatus*, a ubiquitous environmental mold, enters the lungs and causes various diseases, ranging from bronchial asthma to invasive aspergillosis. Treatment for aspergillosis requires prolonged intravenous antifungal therapy, often accompanied by severe side effects [19,20]. Invasive aspergillosis affects more than 2.1 million people annually and is associated with a mortality rate exceeding 85% [4].

Fungal resistance may develop through the alteration or overexpression of drug targets, activation of efflux pumps, reduced cellular permeability, remodeling of the cell wall and membrane, and changes in stress-response pathways. Fungal biofilms, which are encased in an extracellular mannan–glucan matrix that sequesters antifungals, are formed on medical devices and mucosal surfaces; they reduce treatment efficacy and contribute to the development of resistance [21,22,23]. Factors contributing to the spread of resistant and thermotolerant fungal strains include climate change, overuse and inappropriate prescribing of antifungal medications, particularly in pediatric and COVID-19 patients, and widespread application of azole fungicides in agriculture [24,25,26]. Assessing the prevalence of fungal resistance is challenging due to variations in testing methods and interpretation criteria among countries and medical centers. Even CLSI and EUCAST standards differ in their minimum inhibitory concentration (MIC) determinations and clinical breakpoints. It is important to distinguish clinical breakpoints, which are based on clinical outcome data and are intended to guide therapeutic decisions, from epidemiological cutoff values (ECOFFs/ECVs), which are derived solely from MIC distributions of wild-type populations and serve to detect resistant isolates without predicting clinical efficacy. Additionally, clinical breakpoints are not established for specific pathogen–antifungal combinations, in particular, for *A. fumigatus* and echinocandins [27,28,29,30,31]. EUCAST breakpoints for *C. auris* were officially approved for only three antifungal drugs in mid-2025.

A key reason fungal infections remain a major public health concern is that the available antifungal drugs do not meet clinical needs, as noted in a 2025 WHO statement [32]. In contrast to the extensive arsenal of antibacterial agents, only four major therapeutic classes are available to treat infections caused by WHO critical priority fungi: azoles, polyenes, echinocandins, and the antimetabolite flucytosine. Ciclopirox, a member of the hydroxypyridone group, is also used in some cases. Most of these medications have significant limitations, such as low solubility, high toxicity, a narrow therapeutic window, drug–drug interactions, the requirement for long-term administration in a hospital setting, and the necessity to monitor drug concentrations in patients’ bloodstreams [33,34,35]. The fungistatic, rather than fungicidal, action of antimycotics can lead to chronic infection and disease recurrence. Furthermore, few antimycotics are effective against fungal biofilms [36]. Despite the challenging landscape of antifungal therapy, the pace of novel drug development and commercialization remains sluggish. Over the past decade, the Food and Drug Administration (FDA) has approved only three novel antifungals, while, as of 2024, three additional novel antifungal agents were in phase III clinical development [32].

All these facts, combined with the spread of resistant WHO critical priority fungi, create an urgent need to search for new, effective, and safe antifungal agents. Promising antifungal drugs should possess novel mechanisms of action, be effective against resistant and biofilm-associated forms, exhibit high selectivity, and have a favorable safety profile. Key approaches include searching for agents that act on previously unexploited molecular targets or that enhance existing therapies and reduce the risk of resistance. One of the major barriers to the commercialization of new drugs is the rapid development of fungal resistance, partly due to cross-resistance [37]. Therefore, the evaluation of new antifungal agents should consider not only efficacy and safety but also the dynamics of resistance acquisition and the underlying mechanisms.

This review comprehensively surveys conventional antimycotics and novel antifungals, with a focus on the mechanisms underlying resistance in WHO critical priority fungi. We discuss the main strategies currently employed to overcome fungal resistance, with particular emphasis on antifungal peptides (AFPs) of various origins and their synthetic analogs effective against critical priority fungi. Furthermore, we examine the diversity of molecular targets of AFPs and assess the development of fungal resistance to these compounds to determine their potential as prototypes for new antifungal drugs.

## 2. Conventional and Novel Antifungals, and Resistance in WHO Critical Priority Fungi

Recent data indicate that WHO critical priority fungi are developing resistance to all four classes of antifungal agents currently used in clinical practice to treat superficial and invasive mycoses [12,38,39]. This is a matter of major concern that restricts therapeutic options and worsens clinical outcomes [40,41]. Antifungal agents, their targets, clinical applications, and the molecular mechanisms of resistance are detailed below (Table 1).

### 2.1. Azoles

Azoles possess activity against fungi of the *Candida*, *Aspergillus* and *Cryptococcus* genera and are the drugs of choice in many clinical cases, including invasive aspergillosis; yet this class is primarily fungistatic and associated with severe side effects [50].

*C. auris* and *C. albicans* use broadly similar molecular strategies to evade azole action (Table 1), yet the clinical and epidemiological patterns of resistance in these species differ substantially [44]. Resistance in *C. albicans* has developed largely as a result of prolonged azole exposure. It is responsible for recurrent and breakthrough infections and relies on transcriptional rewiring [45]. For example, data from the Northwestern Federal District of Russia show a strong upward trend in resistance to fluconazole and voriconazole increased from 0 to 2% in 2009–2013 to 36.6% and 34.5% in 2019, respectively, and exceeded 46% for both drugs in 2020–2021 [58]. *C. auris* isolates typically show reduced susceptibility to fluconazole (≥32 mg/L), and most of them carry additional resistance mutations in the *ERG11* gene [59]. *C. auris* is characterized by genomic instability, manifested as changes in the copy number of specific genomic regions and accumulation of mutations [60].

Clinical data on azole resistance in cryptococcal meningitis show considerable variation. For instance, in 2018, a systematic analysis of 29 studies, including 4995 clinical *Cryptococcus* spp. isolates, found a mean fluconazole resistance rate of 12.1% [18]. Point mutations affecting ergosterol biosynthesis, hypermutable states due to DNA repair defects, and increased expression of transporters and stress-response proteins promote *Cr. neoformans* resistance (Table 1) [42,60]. Additionally, clinical isolates of *Cr. neoformans* frequently exhibit heteroresistance to fluconazole, wherein small subpopulations acquire chromosomal duplications and ploidy changes that upregulate drug targets and efflux systems, ensuring survival at high drug concentrations [59].

Azole-resistant *A. fumigatus* isolates are increasingly encountered in clinical settings, compromising first-line azole efficacy, correlating with elevated mortality and therapeutic failure, and making azole monotherapy less reliable while prompting earlier use of alternative agents or combination regimens. For example, in an international prospective study published in 2015, the overall prevalence of azole-resistant *A. fumigatus* isolates was 3.2%. *A. fumigatus* resistance can arise both from prolonged therapy and from environmental exposure to azole fungicides, involving conserved mechanisms such as target-site alterations and efflux pumps, along with regulatory and metabolic reprogramming that affects transcriptional networks, mitochondrial function, and redox homeostasis (Table 1) [47,59,61,62].

### 2.2. Polyenes

Among polyenes, AmB exhibits fungicidal activity and is used for the treatment of life-threatening mycoses including severe and moderate cryptococcal meningitis, candidiasis, and all forms of invasive aspergillosis. However, it is characterized by high toxicity (nephrotoxicity, hepatotoxicity) and low water solubility [50].

Polyene resistance in *C. albicans* occurs much less frequently than azole resistance and involves alterations in sterol metabolism and its transcriptional control, leading to modified membrane sterol composition. Furthermore, the activation of stress response pathways can promote fungal cell viability, thereby contributing to the development of polyene resistance in *C. albicans* (Table 1) [50,59,60].

Polyene resistance is also less frequently observed in *C. auris*, but contributes to its multidrug-resistant phenotype. According to a 2024 systematic review, resistance among *C. auris* isolates ranges from 87 to 100% for fluconazole, 28–98% for voriconazole, 8–35% for AmB, and 0–8% for echinocandins [14]. *C. auris* isolates are generally significantly less susceptible to AmB than other *Candida* species, a feature largely attributed to differences in membrane sterol composition, adaptive mitochondrial responses, and altered redox homeostasis. Genetic mutations or dysregulation of the ergosterol biosynthesis pathway underlie polyene resistance development in *C. auris* [46,50].

AmB resistance in *Cr. neoformans* is considered exceptionally rare and is generally attributed to alterations in ergosterol biosynthesis (Table 1). At the same time, host-specific conditions profoundly influence AmB tolerance, highlighting the need for alternative or combination therapy: during pulmonary infection, *Cr. neoformans* may adopt a quiescent state that withstands AmB exposure, whereas in the central nervous system, elevated glucose levels activate glucose repression signaling in fungal cells, and remodel their membrane lipid composition [42,50,63].

*A. fumigatus* rarely develops resistance to AmB, and its molecular basis is still poorly understood. Current evidence indicates that changes in membrane lipid composition, sterol metabolism, and stress response pathways may modulate AmB susceptibility [48,49].

### 2.3. Echinocandins

Echinocandins act fungicidally against most *Candida* species and are first-line agents for the treatment of candidemia and invasive candidiasis when other therapies are ineffective or unsuitable for the patient [32,53]. Echinocandin resistance is rare in *C. albicans* [54] and in *C. auris*, but clinically concerning for the latter, as echinocandins are the primary therapy option for infections caused by this pathogen. Resistance in both cases typically arises from mutations affecting the catalytic subunit of glucan synthase (Table 1) [46].

Echinocandins are ineffective against *Cr. neoformans.* This fungus displays intrinsic resistance to echinocandins, which is not due to alterations in the target enzyme, but rather to the large polysaccharide capsule and a unique cell wall architecture with elevated levels of β-1,6-glucan and chitosan [64,65] as well as to the activation of protective signaling pathways that regulate membrane homeostasis, calcium balance, and cell wall remodeling [42,61,66]. Analysis of libraries for echinocandin-sensitive *Cr. neoformans* mutants revealed that loss or dysregulation of *CDC50* (encoding the β-subunit of the lipid flippase) enhances caspofungin penetration into fungal cells [51,52].

In invasive aspergillosis, echinocandins are combined with polyenes or azoles to attain clinically meaningful synergistic effects, as they are only fungistatic against *Aspergillus* spp. This is due to the fact that β-1,3-glucan synthesis inhibition across all fungal species, but especially in *Aspergillus* spp., elicits a compensatory upregulation of chitin biosynthesis and engagement of signaling pathways that preserve cell wall integrity [67]. Currently, echinocandin resistance in *A. fumigatus* is considered uncommon and is thought to arise from both mutations in the genes encoding the drug target and modifications in the lipid microenvironment of the target enzyme [47,59].

### 2.4. Nucleoside Analogs, Antimetabolites

5-FC exerts a fungistatic effect and is not used as monotherapy due to its narrow therapeutic window, significant hepatotoxicity, and the rapid development of resistance, but it is a component of combination therapy for invasive mycoses, primarily cryptococcal infections in combination with AmB [59].

The key pathways governing 5-FC transport and metabolism are highly conserved between *C. albicans* and *Cr. neoformans*, resulting in broadly analogous resistance mechanisms driven by deficiencies in drug uptake and its intracellular activation (Table 1) [53]. 5-FC resistance in *C. auris* is largely due to species-specific modifications in the pyrimidine salvage pathway that prevent drug activation [60], whereas other *Candida* species also use efflux, stress responses, and transcriptional reprogramming [59,61]. Reduced intracellular 5-FC exposure in *A. fumigatus* is largely governed by pH-dependent regulation of the purine–cytosine permease. Unlike yeast pathogens, *A. fumigatus* employs environmentally controlled drug uptake mechanisms, representing a species-specific strategy for limiting 5-FC access to its intracellular metabolic machinery [60].

### 2.5. Hydroxypyridones

Along with the four main classes, ciclopirox is the only hydroxypyridone employed in the treatment of vaginal candidiasis caused by *C. albicans*. This agent also exhibits antiviral and antitumor activities [68]. Although ciclopirox was approved as a topical antifungal agent more than two decades ago, its mechanism of action remains incompletely understood. Ciclopirox is capable of chelating polyvalent metal cations (Fe^3+^, Al^3+^) and inhibiting metal-dependent enzymes such as cytochromes, catalases, and peroxidases, thereby disrupting mitochondrial function, energy production, and membrane transport [69]. Fungal resistance to ciclopirox has not been observed, even after decades of use in treating the relevant diseases [70].

### 2.6. Novel Antifungals

The agents discussed below illustrate the main directions of antifungal drug development during 2021–2026. Three compounds (ibrexafungerp, oteseconazole, and rezafungin) received FDA approval during this period, whereas fosmanogepix and olorofim remained investigational agents in phase III development; the phase III program of opelconazole was terminated in 2026. These agents differ considerably in their pharmacological novelty. Oteseconazole, rezafungin, and opelconazole are refined derivatives of established antifungal classes and retain the canonical targets of azoles or echinocandins. Ibrexafungerp represents a new structural class of triterpenoid glucan synthase inhibitors, yet it targets the same enzyme as echinocandins. By contrast, fosmanogepix and olorofim are first-in-class agents targeting previously unexploited molecular patterns.

**Oteseconazole** (VT-1161), a tetrazole antimycotic with markedly greater selectivity for fungal lanosterol 14α-demethylase than for human cytochrome P450 enzymes, was approved by the FDA in 2022 for recurrent vulvovaginal candidiasis in females not of reproductive potential [71,72]. Oteseconazole demonstrates high activity against many fluconazole-resistant *Candida* isolates, but increased MICs for some of them suggest partial cross-resistance [73,74,75]. Reduced susceptibility to oteseconazole is associated with increased drug efflux (Tac1-dependent Cdr1/Cdr2 and Pdr1-dependent Cdr1 overexpression), altered Upc2A-dependent sterol regulation, Erg11 overexpression or substitutions, and a loss-of-function ERG3 mutation [73,74,75].

**Rezafungin** (CD101), a next-generation echinocandin structurally related to anidulafungin, with chemical modifications that increase stability and half-life enabling once-weekly administration, was approved by the FDA in 2023 for candidemia and invasive candidiasis in adults with limited or no alternative treatment options [76,77,78,79]. During serial passage of *Candida* species, reduced susceptibility to rezafungin develops relatively slowly and is accompanied by amino acid substitutions in Fks1 and Fks2 [80]. Some echinocandin-resistant *Candida* isolates also showed reduced susceptibility to rezafungin [81,82].

**Ibrexafungerp** (Fungerp, SCY-078), a triterpenoid glucan synthase inhibitor, received FDA approval for an oral formulation for vulvovaginal candidiasis in 2021 and for reducing recurrent vulvovaginal candidiasis in 2022 [83,84]. Ibrexafungerp shares the same target enzyme as echinocandins but with only partial binding-site overlap. Ibrexafungerp is active against most echinocandin-resistant *Candida* isolates [85,86,87], yet exposure can generate *C. glabrata* mutants with *FKS* mutations (overlapping with echinocandin resistance or ibrexafungerp-specific), with the greatest MIC increases for substitutions near the glucan synthase hotspot regions in *C. albicans* and *C. glabrata* [88,89,90,91].

**Fosmanogepix** (APX001), a phosphate prodrug converted to manogepix (APX001A), belongs to a new class of inhibitors of the fungal inositol acyltransferase Gwt1, which catalyzes inositol acylation in glycosylphosphatidylinositol (GPI)-anchor biosynthesis. Since GPI anchors are essential for surface proteins involved in cell wall assembly, adhesion, hyphal development, and host interactions, Gwt1 inhibition disrupts mannoprotein localization, cell wall organization, and fungal growth [92,93]. Fosmanogepix is being evaluated in two phase III programs for candidemia and invasive candidiasis or invasive mold infections, respectively [94,95]. Although Gwt1 is distinct from azole, echinocandin, and polyene targets, making direct target-based cross-resistance unlikely [96], shared efflux mechanisms may link reduced manogepix susceptibility to azole resistance. Serial passage yielded *Candida* strains with reduced manogepix susceptibility mediated by Gwt1 substitutions (without cross-resistance) and increased efflux [97,98,99,100]. Activating mutations in *TAC1*, *TAC1B*, and *PDR1* reduced manogepix susceptibility via ABC transporters, such as Cdr1, and also conferred fluconazole resistance. In addition, a gain-of-function mutation in *ZCF29* activated *CDR11* and *SNQ2* expression in *C. albicans*, whereas a mitochondrial deletion increased *MDR1* expression in *C. parapsilosis* [98,99,100].

**Olorofim** (F901318), a first-in-class orotomide, targets fungal class II dihydroorotate dehydrogenase (DHODH), involved in de novo pyrimidine biosynthesis. Inhibition of DHODH disrupts DNA/RNA synthesis and fungal growth [101,102]. In June 2026, positive phase III topline results were reported for this agent in patients with invasive aspergillosis for whom azole therapy was unsuitable [103]. No intrinsic olorofim resistance was detected among clinical *A. fumigatus* isolates, but experimental selection yielded resistant variants via DHODH substitutions [104]. The agricultural fungicide ipflufenoquin, sharing the same target, selects *A. fumigatus pyrE* (the gene encoding DHODH) mutants cross-resistant to both agents [105]; additionally, multi-azole-resistant strains with elevated mutation rates may accelerate resistance to olorofim [106]. Thus, resistance to new drug classes could arise before clinical deployment through cross-selection by structurally or functionally related compounds.

**Opelconazole** (PC945), a triazole CYP51 inhibitor designed for inhaled administration in pulmonary aspergillosis [107,108], had its phase III trial terminated in 2026 after an interim analysis revealed an unfavorable efficacy and clinical outcome signal, with numerically lower favorable response rates and higher mortality in the opelconazole group versus the control group. At the time, no patient deaths were attributed to the drug in this blinded study [109]. The drug was highly active against azole-susceptible *A. fumigatus* isolates, whereas strains with *cyp51A* mutations, including TR34/L98H and TR46/Y121F/T289A, showed reduced susceptibility, suggesting possible cross-resistance with other azoles [110].

Thus, azole resistance, driven by ERG11/CYP51 mutations and efflux pump overexpression, is the most clinically significant issue across all four critical priority fungi. Echinocandin resistance via FKS hotspot mutations threatens *C. albicans*, *C. auris*, and *A. fumigatus*, while *Cr. neoformans* is intrinsically resistant. Polyene resistance is rare but has been described for all four species, although its mechanisms in *A. fumigatus* remain unclear. The 5-FC resistance limits its monotherapy use. Resistance also emerges to novel antifungals and the most significant risks involve cross-resistance between fosmanogepix and azoles via efflux pump activation, between rezafungin/ibrexafungerp and echinocandins via FKS mutations, and between olorofim and the agricultural fungicide ipflufenoquin. Multidrug-resistant *C. auris* remains the greatest clinical threat, stressing the need for new antifungals and rapid diagnostics.

## 3. Strategies to Overcome Resistance in WHO Critical Priority Fungi

**Figure 1:**
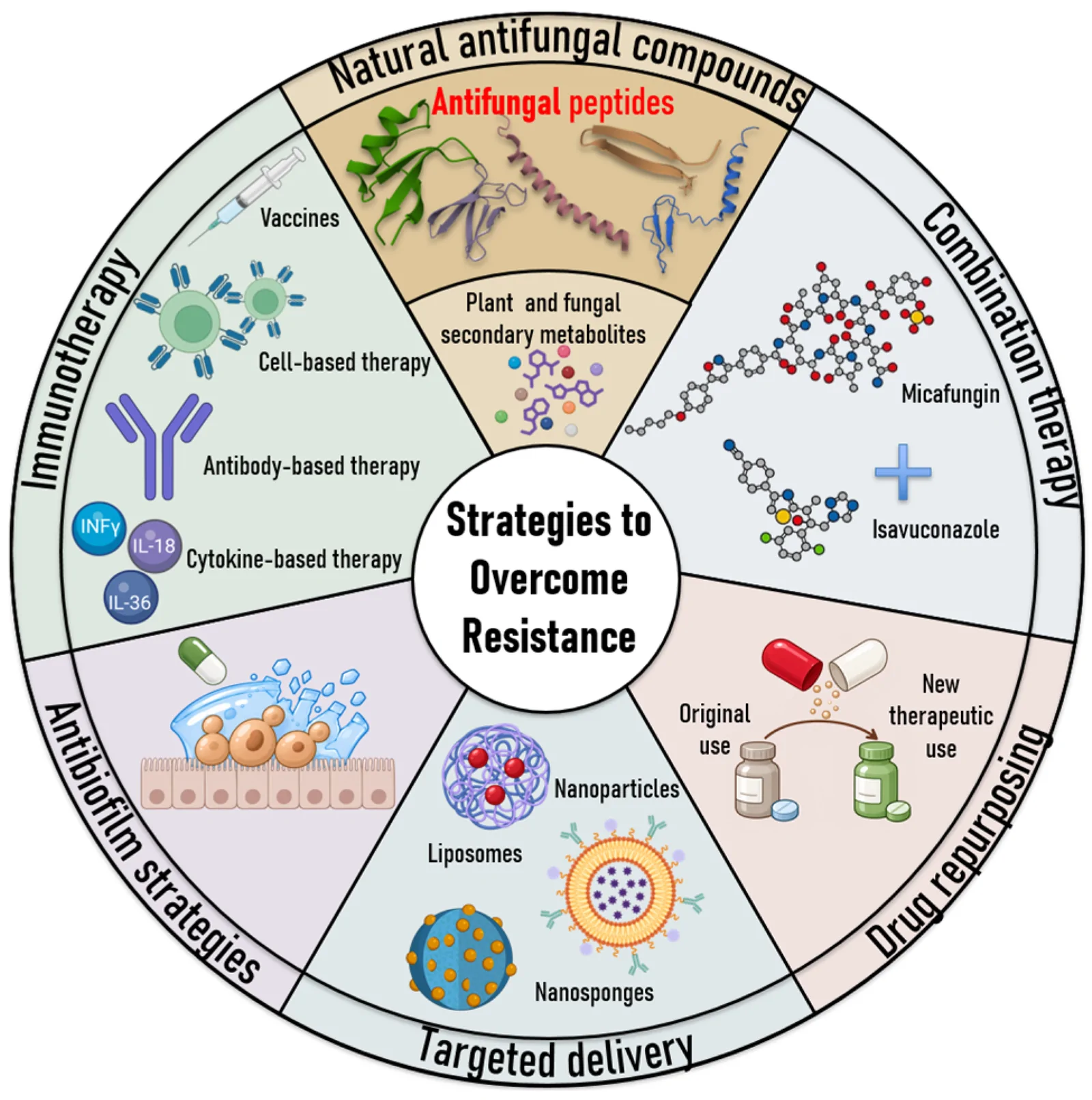
Strategies to overcome resistance in WHO critical priority fungi, including antifungal peptides.

To date, various strategies have been proposed to overcome resistance in WHO critical priority fungi, including combination therapy, drug repurposing, targeted delivery, anti-biofilm approaches, immunotherapy, and the use of natural antifungal compounds (Figure 1, Table 2).

### 3.1. Combination Therapy

A promising approach is combination therapy using drugs with different mechanisms of action to reduce the dose of each antifungal agent, thereby decreasing toxicity and the risk of resistance. Some combinations are already used for the treatment of fungal diseases. Synergistic effects have been described for azoles and echinocandins or polyenes [111]. Triazole–echinocandin combinations are effective against fungi of the *Candida* genus [112,113], particularly the isavuconazole–micafungin combination against multidrug-resistant *C. auris* [113]. 5-FC combined with echinocandins or AmB effectively suppresses the growth of both echinocandin- and polyene-resistant *C. auris* strains. The combination of AmB with 5-FC is recommended as the induction strategy for cryptococcal meningitis, central nervous system infections, and *Candida* endocarditis. The combination of AmB with fluconazole is considered when 5-FC is unavailable [112,113,114].

### 3.2. Drug Repurposing

Drug repurposing is a strategy that uses already approved drugs for new therapeutic indications to reduce the time and cost of antifungal development [115]. Certain antibiotics also possess antifungal activity in both in vitro and in vivo studies. Colistin is active against multidrug-resistant *Candida* and *Cryptococcus* strains possibly due to a disruption of fungal membrane permeability. Polymyxin B disrupts *Cr. neoformans* and *A. fumigatus* cell membranes by binding to anionic lipids. Erythromycin enhances the efficacy of AmB against *C. albicans*, *C. auris*, and *Cr. neoformans*, inhibiting mitochondrial protein synthesis and efflux pump activity, and reducing ergosterol levels [116,117]. Anticancer agents, such as tamoxifen, are also active against *C. albicans* and *Cr. neoformans.* Statins inhibit the growth of *Candida*, *Cr. neoformans*, and *A. fumigatus* and also modulate host immune responses. Ibuprofen kills *Cryptococcus* cells through ROS production, membrane damage, and activation of the high-osmolarity glycerol pathway. Neuroleptics (haloperidol, trifluperidol, and sertraline) also exhibit activity against critical priority fungi [118].

### 3.3. Targeted Delivery

Various nanocarriers (liposomes, nanoemulsions, nanosponges, nanostructured lipid carriers, polymeric nanoparticles, micelles, dendrimers) have been investigated to reduce toxicity and enhance the solubility, stability, and targeting efficiency of antifungal drugs [119]. Currently, liposomal AmB (AmBisome^®^, Fungisome^®^) remains the only commercially available, clinically approved antifungal formulation. Liposomal formulations offer superior safety, with markedly reduced systemic toxicity and a lower incidence of adverse effects [120]. Targeted AmB liposomes functionalized with DC-SIGN constructs, fusing the carbohydrate recognition domain with different neck repeats, were developed. These liposomes bind to exopolysaccharides of *A. fumigatus*, *C. albicans*, and *Cr. neoformans* and exhibit high efficacy in murine models of invasive candidiasis and pulmonary aspergillosis [121]. Niosomes, nonionic surfactant vesicles, are administered via various routes, providing prolonged release, improved bioavailability for poorly soluble drugs, and protection from premature degradation. Nystatin-loaded niosomes have been successfully developed, demonstrating reduced nephro- and hepatotoxicity as well as enhanced therapeutic efficacy against *C. albicans* in in vivo models [122].

### 3.4. Antibiofilm Strategies

A key virulence factor of pathogenic fungi, including *Candida*, *Aspergillus*, and *Cryptococcus*, is the ability to form biofilms [123,124]. Biofilm resistance is driven by the extracellular matrix, constitutive efflux pump activity, and metabolically inactive persister cells [123]. Biofilms often result in persistent or recurrent infections, requiring higher antifungal doses [125]. The main strategy to overcome biofilm resistance is combination therapy, which achieves synergistic effects by pairing conventional antimycotics (azoles, AmB, echinocandins) with non-antimycotic drugs, natural compounds, or other antifungal agents. Promising combinations include calcineurin inhibitors, statins, antibiotics, and drugs from other therapeutic classes, which enhance efficacy by disrupting the extracellular matrix, inhibiting secreted fungal enzymes, affecting the ergosterol biosynthesis pathway, attenuating stress responses, and blocking efflux pumps [59,126].

### 3.5. Immunotherapy

Immune-based therapy is another fundamentally important approach to overcoming resistance. Besides vaccines, cell-based, cytokine-based, and antibody-based immunotherapy strategies are currently under investigation.

#### 3.5.1. Vaccines

Currently, there are no approved antifungal vaccines for clinical use. However, several candidates are at the preclinical testing stage or have reached the stage of human trials. For example, the vaccine platform NDV-3/NDV-3A showed efficacy against recurrent vulvovaginal candidiasis in women in a phase Ib/IIa trial [127]. The conjugate pentavalent vaccine Candi5V is undergoing phase I/II human testing against vulvovaginitis [128]. The virosomal vaccine PEV7 demonstrated efficacy in phase I clinical trials [129]. The anti-*A. fumigatus* vaccine VesiVax^®^ Af3/9 improved survival in a neutropenic mouse model [130]. The anti-cryptococcal vaccine Cda2-Pep1 GP conferred protection against a highly virulent strain in BALB/c and C57BL/6 mice [131]. “Classic” vaccines based on live/heat-inactivated antigens are also under development [132]. The heat-killed fbp1Δ mutant *Cr. neoformans* is a promising option, affording cross-reactivity to *Cr. gattii* and *A. fumigatus* strains [133,134,135]. Administration of ΔsglA *A. fumigatus* conidia afforded full protection against the wild-type fungus [136]. The CDA1-LNP mRNA vaccine against *Cr. neoformans* ensured the survival of the majority of infected mice [137].

#### 3.5.2. Cell-Based Therapy

Cell-based therapies include granulocytes, dendritic cells (DCs), natural killers (NKs), T lymphocytes as well as modified CAR-T and CAR-NK cell therapies [138,139]. Granulocyte transfusion is proposed to enhance host immunity in neutropenic patients with invasive mycoses [140]. However, findings are inconsistent, and randomized trials often do not show a survival benefit [141,142]. DCs stimulated with *Aspergillus* spp., *C. albicans*, and *Cr. gattii* antigens induce protective immune responses [143,144,145]. Transferring activated NK cells from wild-type mice to IFN-γ-deficient or wild-type neutropenic mice relieves aspergillosis [146,147]. Transfusion of *A. fumigatus*-specific T cells enhances protection in preclinical models [148,149,150] and small clinical trials [151,152]. CAR-NK-92 cells targeting *A. fumigatus* reduce fungal growth and cell count in immunodeficient mice [153], while SRCD5CAR-based CAR-NK cells demonstrate efficacy against *C. albicans* and *Cr. neoformans* in vitro and in vivo [154]. However, clinical trials of primary and modified T or NK cells are currently lacking [139].

#### 3.5.3. Cytokine-Based Therapy

The applicability of various cytokines in the treatment of fungal infections has been demonstrated. Granulocyte and macrophage colony-stimulating factors increase the survival rate in animal models of *A. fumigatus* infection [155,156]. IFN-γ induces antifungal immunity in patients with invasive *Cryptococcus*, *Candida*, or *Aspergillus* infections [157,158,159]. IL-36 and IL-18 enhance the host immune response against various fungal infections [160,161].

#### 3.5.4. Antibody-Based Therapy

Immunotherapy using monoclonal antibodies (mAbs) has shown promising results against invasive mycoses in preclinical studies, but data on clinical testing are limited. The HSP90-specific antibody efungumab has shown high efficacy against aspergillosis and candidiasis, and has demonstrated improved efficacy in combination with AmB, although it is not approved for medical use. Antibodies against conserved fungal antigens and immunomodulatory antibodies (anti-CD40, anti-PD-1/PD-L1) also provide antifungal protection in murine models [138].

### 3.6. Natural Antifungal Compounds

Natural compounds such as plant and fungal secondary metabolites and antimicrobial peptides (AMPs) from various sources are seen as promising alternatives due to their structural diversity and multiple mechanisms of action [162]. Fatty acids and their methyl esters exhibit antifungal properties by disrupting the cell membrane, inhibiting ergosterol synthesis, and suppressing virulence factors. 6-Nonadecenoic acid from *Pentagonia gigantifolia* is active against *C. albicans*, *A. fumigatus*, and *Cr. neoformans*; lauric, myristic, and palmitic acids also exhibit antifungal activity [163]. Plant terpenoids, in particular camphor and eucalyptol, possess activity against planktonic cells and biofilms of different *Candida* species. Camphor also reduces ROS production, which can cause epithelial cell damage during infection [164]. Many plant flavonoids inhibit fungal growth, affecting the expression of genes encoding efflux pumps (*CDR1*) and an ergosterol biosynthetic enzyme (*ERG11*). Isobavachalcone from *Maclura tinctoria* is active against *C. albicans* and *Cr. neoformans*. Isoquercitrin inhibits *C. albicans* growth and reduces fungal biofilm formation [165]. Saponins such as phytolaccoside B from *Phytolacca tetramera* have a unique mechanism for stimulating chitin synthesis, which leads to cell wall thickening [166]. Endophytic fungi (*Aspergillus*, *Pestalotiopsis*, *Mycosphaerella*) are a promising source of metabolites with high activity against *C. albicans* and *Cr. neoformans*, for example, cryptocandin and monoterpenoids, which inhibit cell wall synthesis [167].

Thus, among strategies to overcome resistance in critical priority fungi described above (Table 2), combination therapy using antimycotics from different classes and targeted polyene delivery, which markedly reduce their toxicity, are already in clinical use. Extensive in vivo data and numerous clinical trials support the high potential of immunotherapy, including vaccines, cell-based therapies, and antibody- and cytokine-based agents. In contrast, limited translational evidence for drug repurposing, antibiofilm strategies, and natural antifungal compounds underscores the need for their further preclinical and clinical investigation.

AMPs are ancient, evolutionarily conserved effector molecules of innate immunity and interspecies competition, found across bacteria, archaea, fungi, plants, and animals. In multicellular organisms, they provide the first line of defense against invading pathogens (bacteria, fungi, viruses). For microbes, AMPs serve as weapons in the struggle for nutrient sources. Besides their direct antimicrobial effects, AMPs possess regulatory functions, being involved in immunomodulation in multicellular organisms and in quorum sensing in unicellular organisms. Their ubiquity throughout the tree of life offers a rich, largely untapped reservoir for novel antimicrobials. Currently, numerous AMPs have demonstrated activity against fungal pathogens of critical priority in vitro and in vivo. These AFPs are considered promising alternatives to conventional antimycotics, owing to their structural diversity, unique mechanisms of action, low risk of resistance development in repeated-exposure experiments, and capacity to target refractory biofilm-associated infections, as elaborated in detail in the subsequent sections.

## 4. Antifungal Peptides Active Against WHO Critical Priority Fungi

Here we deliberately focused on experimental studies indexed in PubMed that investigated AFPs active against *C. albicans*, *C. auris*, *A. fumigatus* and *Cr. neoformans* included in the 2022 WHO critical priority fungi list. The temporal scope covered approximately the mid-1990s to the present, corresponding to the period of most intensive research on AMPs. The review encompassed natural AFPs from diverse sources, along with selected semi-synthetic derivatives. Only molecules up to 100 amino-acid residues in length that are products of ribosomal biosynthesis (in some cases with post-translational modifications) were considered. Peptides showing potent activity against at least one critical priority pathogen (MIC ≤ 32 µM, determined by standard methods) were selected for inclusion. Additional criteria included published data on AFP activity against resistant clinical isolates, antibiofilm effects, efficacy in animal models of superficial and systemic mycoses, and clinical trial results.

**Figure 2:**
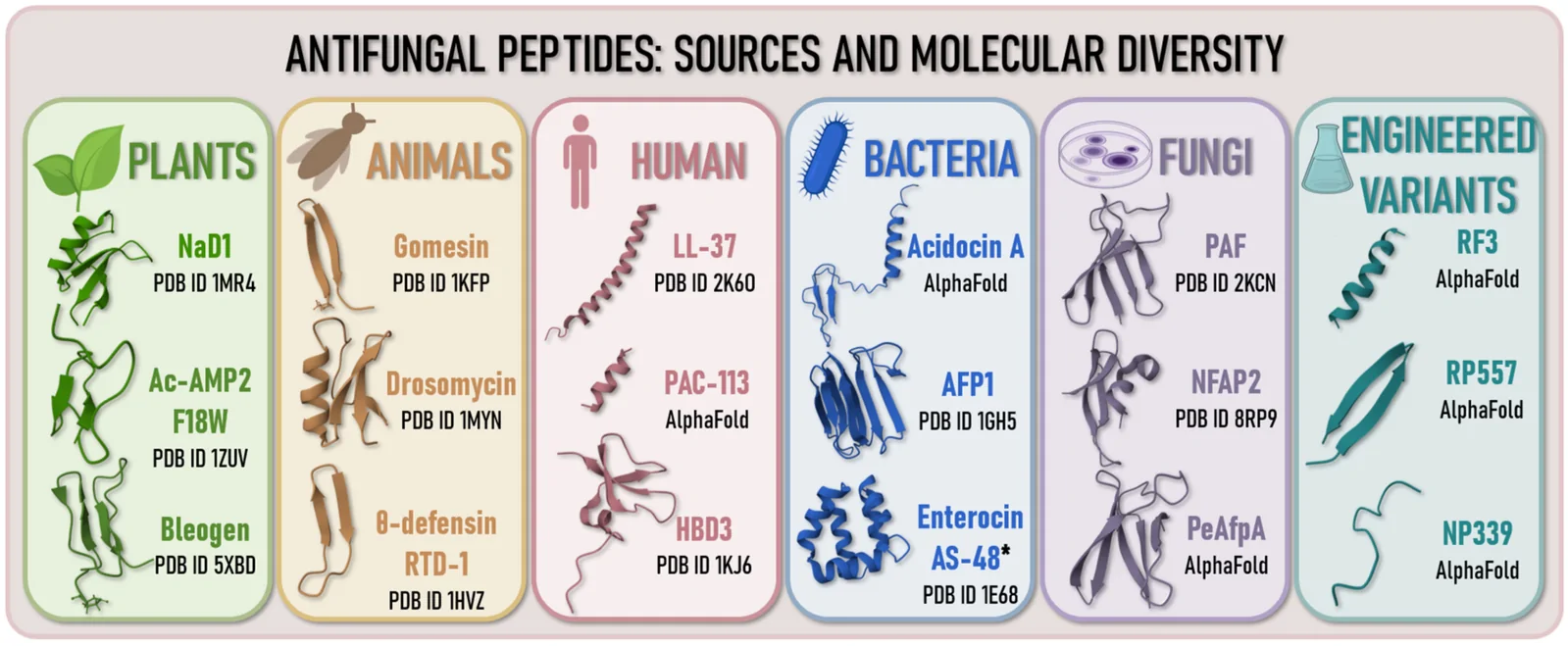
Three-dimensional structures of natural AFPs and their engineered variants. Shown are peptides that meet the criteria outlined at the beginning of Section 4 and possess reliably demonstrated activity against WHO critical priority fungi. Preference was given to AFPs with experimentally determined structures deposited in the Worldwide Protein Data Bank. Otherwise, AlphaFold3-predicted structures (https://alphafoldserver.com (accessed on 8 September 2026)) are shown. * Only the short linearized fragment of enterocin AS-48 (peptide no. 32), but not the peptide itself, shows antifungal activity.

Bacteria, fungi, plants, animals, and humans are sources of AFPs that are effective against critical priority fungi. These AFPs encompass multiple structural classes, ranging from α-helical and β-sheet peptides to those with a mixed α/β fold, many of which are further stabilized by disulfide bridges. The structural diversity of AFPs determines the variety of their properties, including mechanisms of action, antifungal spectrum and potency, cytotoxicity toward human cells, and resistance to proteolysis. Figure 2 illustrates the structural diversity of AFPs, using representative examples from the main peptide classes described in the text.

### 4.1. Plants

Fungi are the main natural pathogens of plants. Therefore, it is not surprising that many plant AMPs target fungi rather than bacteria specifically. Many of these peptides are active not only against phytopathogenic fungi but also against fungi that pose a threat to humans, including species of WHO critical priority.

Plant **defensins** are small cationic peptides (45–54 aa) whose tertiary structure consists of one α-helix and three antiparallel β-strands that are linked by four disulfide bonds forming a cysteine-stabilized αβ (CSαβ) motif [168]. A number of plant defensins have been shown to exhibit high activity against fungi of the *Candida* genus. For example, **NaD1** from *Nicotiana alata* flowers (RE**C**KTESNTFPGI**C**ITKPP**C**RKA**C**ISEKFTDGH**C**SKILRR**C**L**C**TKP**C**; 47 aa; 5.3 kDa; pI 8.5) demonstrates equally high activity against susceptible and resistant strains of *C. albicans* (MIC 6.25 μM) and acts synergistically with caspofungin and endogenous AMPs (human β-defensin 2 (HBD2) and cathelicidin LL-37) [169]. The peptide inhibited fungal cell adhesion to a monolayer of Caco-2 cells, mimicking the human intestinal epithelial barrier, and biofilm formation, but exhibited cytotoxic properties. Meanwhile, its analogs, which have substitutions of amino acids in the A5 loop with arginine, possessed anti-*Candida* activity but exhibited no hemolytic effects and low cytotoxicity [170]. RsAFP2 from *Raphanus sativus* prevents *C. albicans* biofilm formation, acts synergistically with caspofungin and AmB, does not exhibit cytotoxic properties and prevents the development of disseminated candidiasis in a mouse model [168,171]. HsAFP1 from *Heuchera sanguinea* seeds also possesses antifungal and antibiofilm activities [172]. Pronounced antifungal activity against *C. albicans* and other fungi of this genus has also been shown for many other plant defensins, including DmAMP1 from *Dahlia merckii*, PvD1 from *Phaseolus vulgaris*, AFP1 from *Raphanus sativus*, NbD6 from *Nicotiana benthamiana*, OsAFP1 from *Oryza sativa*, ZmD32 from *Zea mays*, So-D2 from *Spinacia oleracea*, EcgDf1 from *Erythrina crista-galli* [168]. Defensin-like peptides from *Medicago truncatula* known as nodule-specific cysteine-rich (NCR) peptides and their derivatives demonstrate activity against different fungi of the *Candida* genus (MIC 1.42–10.5 µM) as well as against *Cr. neoformans* (MIC 0.39 μM for **X1-NCR247C** (RPLNFKMLRFWGQQQ**C**RRPLY**C**RRR; 25 aa; 3.3 kDa; pI 12)) [173]. Plant defensins typically have multiple targets, but their antifungal activity is largely attributed to interaction with universal (phosphatidic acid (PA), phosphatidylinositol 4,5-bisphosphate (PI(4,5)P_2_) or fungus-specific (mannosyl-diinositol-phosphorylceramide (M(IP)_2_C), glucosylceramides (GlCers)) lipids.

**Hevein-like peptides** are small cysteine-rich AFPs (29–45 aa) whose structure contains a conserved chitin-binding site (SXFGY/SXYGY). Their spatial structure is usually represented by three antiparallel β-sheets and a short α-helix, and stabilized by 3–5 disulfide bonds. It has been shown that the modified hevein-like peptide Ac-AMP2 from *Amaranthus caudatus* (**mAc-AMP2**; VGE**C**VRGR**C**PSGA**CC**SQWGY**C**GKGPKY**C**GR; 30 aa; 3.2 kDa; pI 8.4) possesses activity against susceptible and resistant strains of fungi of the genus *Candida* at nanomolar concentrations (MIC 0.39 μM), exhibits anti-adherent and antibiofilm properties, but does not demonstrate hemolytic and cytotoxic activities [174]. Chitin-binding peptide **Cc-Hev** from *Capsicum chinense* exhibits activity against *C. albicans* [175].

Activity against fungi of critical priority has been demonstrated for some representatives of other classes [176]. **MCh-AMP1**, an antifungal peptide from *Matricaria chamomilla* L., whose structure does not have significant similarity with known classes of plant AMPs (LSVKAFTGLQLRGV**C**GLEVKARG; 23 aa; 2.4 kDa; pI 10.7), demonstrates activity against *C. albicans* and *A. fumigatus* with an MIC value of 6.66 μM. It is worth noting that the peptide has a fungicidal effect on both types of fungi, but exhibits cytotoxic and hemolytic effects [177]. **Bleogen pB1** from *Pereskia bleo* (Z**C**KPNGAK**C**TEISIPP**CC**SNF**C**LRYAGQKSGT**C**ANR, where Z represents pyroglutamic acid; 36 aa; 4 kDa; pI 8.1) sharing sequence similarity with plant knottins, is non-toxic to mammalian cells but acts effectively against *C. albicans* (MIC 5 μM) [178]. The fraction containing two **vicilin-like peptides** from *Capsicum baccatum* (4 and 8 kDa), which have structural similarity to the vicilins (7S storage globulins), exhibits activity against *C. albicans* [176,179]. A small peptide **Tn-AFP1** (LM**C**THPLD**C**SN; 11 aa; 1.2 kDa; pI 4.9) purified from *Trapa natans*, inhibits *C. tropicalis* growth (MIC 26 μM) and disrupts biofilm formation but causes lysis of red blood cells at the MIC [180].

### 4.2. Animals

Among AMPs of animal origin, both strictly antibacterial and specialized antifungal peptides, as well as broad-spectrum peptides, are widely represented. According to their structure, they are classified into (1) Cys-containing, forming disulfide bonds, (2) linear (α-helical) and (3) enriched with certain amino acid residues (Pro, Gly, Trp, etc.).

β-hairpin AMPs are a subclass of Cys-containing peptides that form two antiparallel β-strands connected by a β-turn and stabilized by one or two disulfide bonds. **Tachyplesin I** (KW**C**FRV**C**YRGI**C**YRR**C**R-NH_2_; 17 aa; 2.3 kDa; pI 10), isolated from the hemocytes of the horseshoe crab *Tachypleus tridentatus*, inhibits *C. albicans* growth (MIC_90_ 7 µM), suppresses biofilm formation and, to a lesser extent, eradicates preformed biofilms [181]. Recently, a tachyplesin-family peptide, **QS18** (Q**C**FKV**C**FRKR**C**FTK**C**SRS; 18 aa; 2.2 kDa; pI 10.4), which inhibits *Cr. neoformans* with MICs of 1.4–2.8 µM, was identified in the venom gland of the tarantula *Chilobrachys liboensis* [182]. The antifungal mechanism of tachyplesins is generally attributed to direct, non-receptor-mediated membranolysis driven by electrostatic attraction to anionic fungal membrane components, followed by hydrophobic insertion and membrane permeabilization.

The β-hairpin peptide **gomesin** (Z**C**RRL**C**YKQR**C**VTY**C**RGR-NH_2_, where Z represents pyroglutamic acid; 18 aa; 2.4 kDa; pI 10.5) produced in the hemocytes of the Brazilian spider *Acanthoscurria gomesiana*, exhibits activity against *C. albicans* and *Cr. neoformans*, acts in synergism with fluconazole, but possesses hemolytic and cytotoxic properties in vitro [183]. At the same time, this peptide has been shown to be effective in murine models of disseminated and vaginal candidiasis, to exhibit immunomodulatory activity, and to be non-toxic to mice [184]. A natural, gomesin-like, His-rich shorter peptide **DsGom** (R**C**HRV**C**YHKH**C**VQY**C**; 15 aa; 2 kDa; pI 8.5) from the spider *Dysdera sylvatica*, displays activity against susceptible and resistant fungi of the *Candida* genus comparable with that of gomesin, but has a significantly better toxicity profile [185].

**Lactoferricin B** (FK**C**RRWQWRMKKLGAPSIT**C**VRRAF; 25 aa; 3.1 kDa; pI 11.8) generated by pepsin cleavage of the N-terminal region of bovine lactoferrin, shows activity against *C. albicans*, including fluconazole-resistant strains (MICs 0.25–130 µM), and *Cr. neoformans* (MIC ~0.2 µM), functions synergistically with azoles, but is not active against filamentous fungi. The fungicidal mechanism centers on the membrane disruption [186,187]. **LfcinB15**, a fragment of lactoferricin B (FK**C**RRWQWRMKKLGA-NH_2_; 15 aa; 2 kDa; pI 12.4), maintains nearly the same level of antifungal activity coupled with low cytotoxicity. It inhibits *Candida* planktonic cells, shows antibiofilm activity and has multiple modes of action, including mitochondrial damage [188].

The **defensin-like** insect peptide **drosomycin** (D**C**LSGRYKGP**C**AVWDNET**C**RRV**C**KEEGRSSGH**C**SPSLK**C**W**C**EG**C**; 44 aa; 4.9 kDa; pI 7.7) from *Drosophila melanogaster* is stabilized by four disulfide bridges and forms one α-helix and three β-strands in “βαββ” configuration [189]. It acts against *A. fumigatus*, inhibiting spore germination and hyphal growth, and causes partial lysis of hyphae. Drosomycin’s precise molecular target remains unresolved, but conserved surface epitopes (the α- and γ-patches) and an exposed “m-loop” have been proposed as the key regions participating in the interaction with the fungal cell. Drosomycin and related peptides have striking structural–functional similarity to the plant defensins described above. Finding drosomycin-like peptides in the fruit nematode *Caenorhabditis remanei* (**cremycins**) has led to the hypothesis on the horizontal gene transfer from plants to ecdysozoans (molting animals) [190]. Cremycins were shown to be active against numerous *C. albicans* strains in µM concentrations.

Another CSαβ defensin-like peptide **heliomicin** (DKLIGS**C**VWGAVNYTSD**C**NGE**C**KRRGYKGGH**C**GSFANVN**C**W**C**ET-NH_2_; 44 aa; 4.8 kDa; pI 7.8), isolated from the hemolymph of the tobacco budworm *Heliothis virescens*, shares sequence and structural similarity to drosomycin-like peptides, but lacks the fourth disulfide bridge linking the N- and C-termini [191,192]. It is also active against *C. albicans* (MICs 2.5–5 µM), *Cr. neoformans* (2.5–5 µM), and *A. fumigatus* (6–12 µM), being more tolerant to high ionic strength of the test medium than drosomycin. Like some plant defensins, heliomicin interacts with fungal GlCers [193]. **ETD151** (DKLIGS**C**VWGAVNYTSN**C**RAE**C**KRRGYKGGH**C**GSFANVN**C**W**C**ET; 44 aa; 4.9 kDa; pI 8.1), a mutant of insect defensin ARD1 from *Archaeoprepona demophon*, is active against susceptible and azole-resistant *A. fumigatus* strains, retains activity in the presence of bronchial mucus, and shows no toxicity towards bronchial cells. GlCers are also possible molecular targets of this peptide [194].

**Termicins** consist of a family of 6-Cys defensin-like peptides from the insects of the order Blattodea (termites and cockroaches), which have a spatial organization similar to that of drosomycin-like peptides and heliomicin [195,196]. **Termicin** (A**C**NFQS**C**WAT**C**QAQHSIYFRRAF**C**DRSQ**C**K**C**VFVRG-NH2; 34 aa; 4.22 kDa; pI 9.0), isolated from the termite *Pseudacanthotermes spiniger*, exhibits activity against *C. albicans* and *Cr. neoformans* at a 2-fold higher concentration than heliomicin. It does not kill spores of *A. fumigatus*, but causes reduced hyphal elongation with increased branching, similar to some plant defensins.

Macrocyclic rhesus macaque **θ-defensin RTD-1** (GF**C**R**C**L**C**RRGV**C**R**C**I**C**TR, wherein the Gly1 is linked through a peptide bond to Arg18; 18 aa; 2.1 kDa pI 8.8) has a β-hairpin structure and is fungicidal against fluconazole/caspofungin-resistant *C. albicans* strains, and active against established fungal biofilms. RTD-1 has been shown to have efficacy in vivo in a murine model of systemic candidiasis, which was associated with fungal clearance and immunomodulatory effects of the peptide [197].

**Dermaseptins** constitute a large superfamily of lysine-rich cationic AMPs, first identified in skin secretions of South American Hylidae frogs. They are typically 24–34 residues long and form an amphipathic α-helix. **Dermaseptin S3** (ALWKNMLKGIGKLAGKAALGAVKKLVGAES; 29 aa; 3 kDa; pI 11) exhibits activity against *C. albicans*, *Cr. neoformans* and *A. fumigatus* (MICs 10, 1 and 20 μM, respectively) [198].

### 4.3. Humans

Numerous studies have shown that human AMPs such as cathelicidins, defensins, and histatins play an important role in innate defense against fungal pathogens and act effectively against critical priority fungi.

Human **cathelicidin LL-37** (LLGDFFRKSKEKIGKEFKRIVQRIKDFLRNLVPRTES; 37 aa; 4.5 kDa, pI 11.2), having an α-helical fold in the presence of lipid membranes, micelles, or hydrophobic solvents, is characterized by a broad spectrum of activity: it effectively acts against bacteria, fungi, viruses, parasites; and it exhibits immunomodulatory effects by augmenting cellular killing capacity, differentiating and recruiting immune cells, regulating the production of pro-inflammatory cytokines, triggering apoptosis, and promoting angiogenesis [199]. LL-37 inhibits mycelial growth and mycelial adhesion of *A. fumigatus* and prevents fungal invasion and destruction of epithelial cells. In a mouse model of pulmonary *A. fumigatus* infection, LL-37 decreased fungal load and pathological damage and reduced production of proinflammatory cytokines [200]. At the same time, it has been observed that LL-37 causes hemolysis and is toxic to different human cell lines [201]. The bovine ortholog **BMAP-28** (GGLRSLGRKILRAWKKYGPIIVPIIRI-NH_2_; 27 aa; 3.1 kDa, pI 12.6) is more effective than LL-37 against planktonically grown vaginal isolates of *C. albicans* and *C. krusei* in synthetic vaginal simulated fluid (MIC range 8–32 μM) and against *Candida* biofilms [202].

**Histatins** are a group of cationic, His-rich peptides found in saliva that play an important role in immune defense within the oral cavity. **Histatin-5** (DSHAKRHHGYKRKFHEKHHSHRGY; 24 aa; 3 kDa, pI 10.8), derived from the longer precursor of histatin-3, has no defined structure in H_2_O but adopts a more helical conformation in dimethyl sulfoxide and aqueous trifluoroethanol. It possesses pronounced fungicidal activity against *C. albicans*, binding to the fungal cell wall followed by intracellular translocation and disruption of mitochondrial functions. Histatin-5 has demonstrated efficacy in murine models of oral and vaginal candidiasis, and its shortened variant, **PAC-113** (AKRHHGYKRKFH; 12 aa; 1.6 kDa, pI 11.5), has completed phase II trials for oral candidiasis treatment (NCT00659971) [203,204].

**Human β-defensins** (HBDs) are small cationic AMPs consisting of 36–47 aa. They are primarily produced by the epithelium of the respiratory, urogenital and gastrointestinal tracts, skin, and mucous membranes, exhibit a broad spectrum of antimicrobial and immunomodulatory activities and play an important role in host immune defense. HBDs are characterized by a highly conserved, compact spatial structure stabilized by three disulfide bonds, resembling that of plant defensins and consisting of a short N-terminal α-helix and three anti-parallel β-strands. HBDs, especially HBD3, (GIINTLQKYY**C**RVRGGR**C**AVLS**C**LPKEEQIGK**C**STRGRK**CC**RRKK; 45 aa; 5.2 kDa, pI 10.5) possess pronounced activity against different *Candida* species (minimal fungicidal concentration (MFC) 2.5 μM for *C. albicans*) [205]. As in the case with certain plant defensins, including tobacco NaD1, the action of HBD2 and HBD3 is mediated by binding to PI(4,5)P_2_ [206]. Nevertheless, HBD3 as well as LL-37 elevates the β-1,3-exoglucanase activity of *C. albicans* Xog1p, resulting in compromised cell-wall integrity and reduced fungal adhesion [207].

**Human α-defensins** are small peptides (3.5–4 kDa) whose tertiary structure is based on a β-sheet stabilized by three disulfide bonds. They are subdivided into human neutrophil peptides (HNP-1–4) and enteric defensins HD5 and HD6, which are expressed in Paneth cells of the small intestine and contribute to the protection of the gastrointestinal tract. **HNP-1** (A**C**Y**C**RIPA**C**IAGERRYGT**C**IYQGRLWAF**CC**; 30 aa; 3.5 kDa; pI 8.1) acts against clinical *C. auris* isolates, triggering both early and late apoptosis and inducing mitochondrial membrane depolarization and cytochrome c release [208]. It inhibits the metabolic activity and decreases the density of growing and mature biofilms of *C. auris*, and downregulates the genes linked to fungal virulence and efflux pumps [209]. **HD6** (AT**C**Y**C**RTGR**C**ATRESLSGV**C**EISGRLYRL**CC**R; 32 aa; 3.6 kDa; pI 8.4) does not affect *C. albicans* growth, but inhibits fungal adhesion to human intestinal epithelial cells and biofilm formation, as well as suppresses fungal invasion [210].

Lactoferrin-derived AFP, **hLF1-11** (GRRRRSVQW**C**A; 11 aa; 1.37 kDa; pI 12.0), corresponds to the first 11 N-terminal residues of human lactoferrin. The peptide exhibits activity against *A. fumigatus*, *C. auris* and *C. albicans*, including a fluconazole-resistant clinical isolate (MICs 5–32 µM), inhibits biofilm formation, acts synergistically with fluconazole, anidulafungin, and caspofungin, demonstrates efficacy in a murine model of disseminated candidiasis and possesses immunomodulatory properties [211,212,213,214,215,216]. hLF1-11 was well tolerated upon intravenous administration in phase I clinical testing [217], but a randomized phase IIa trial in patients with candidemia (NCT00509834) was withdrawn before enrollment by decision of the company AM-Pharma.

### 4.4. Bacteria

Antifungal activity is generally not characteristic of ribosomally synthesized AMPs of bacterial origin (**bacteriocins**), most of which serve in intraspecific competition. The antibacterial activity of bacteriocins is often mediated by selective interactions with such molecular targets as lipid II or mannose-phosphotransferase which are absent in fungi. Nevertheless, antifungal activity has been observed in a number of bacteriocins and their derivatives.

**Acidocin A** (KTYYGTNGVH**C**TKKSLWGKVRLKNVIPGTL**C**RKQSLPIKQDLKILLGWATGAFGKTFH; 58 aa; 6.5 kDa, pI 10.3) [218] and the related shorter peptide **acidocin 8912** (KTHYPTNAWKSLWKGFWESLRYTDGF; 26 aa; 3.15 kDa; pI 8.4) [219], produced by *Lactobacillus acidophilus*, belong to a distinct subfamily of class II bacteriocins. Acidocin A shows moderate cytotoxicity to human cells, inhibits growth of susceptible and azole-resistant strains of *C. albicans* (MICs 4–8 µM), and exerts a fungicidal effect by disrupting membrane permeability and causing fungal cell lysis [220]. Furthermore, it inhibits fungal adhesion and biofilm formation, and reduces cell viability within preformed fungal biofilms [221]. Acidocin 8912 is substantially weaker (MICs 16–32 µM). Both peptides act presumably by non-specific disruption of membrane barrier function.

A circular bacteriocin **enterocin AS-48** linked head-to-tail is produced by *Enterococcus faecalis* [222] and possesses antibacterial activity exclusively, but some of its truncated, linearized, membrane-interacting fragments show antifungal activity against *Cr. neoformans* and *C. albicans* [223]. In particular, **peptide no. 32** (AGWERIAKELKKYIKKGKKVIRAAW; 25 aa; 2.97 kDa; pI 10.5) matches fluconazole’s growth-inhibitory potency while remaining minimally cytotoxic to human keratinocytes. Its mechanism appears to be linked to disruption of multivesicular bodies or the polysaccharide capsule rather than simple membrane lysis [223].

A small extracellular chitin-binding protein **AFP1** (INRTD**C**NENSYLEIHNNEGRDTL**C**FANAGTMPVAIYGVNWVESGNNVVTLQFQRNLSDPRLETITLQKWGSWNPGH-IHEILSIRIY; 86 aa, 9.9 kDa; pI 5.3) produced by *Streptomyces tendae* does not affect the growth of *C. albicans*, but exhibits potent activity against *A. fumigatus*, which is strongly potentiated when combined with the chitin synthase inhibitor nikkomycin Z [224]. AFP1’s structure consists of two antiparallel β-sheets (five and four strands, respectively) packed into a parallel β-sandwich fold and is stabilized by a single disulfide bridge [225].

### 4.5. Fungi

Fungal-derived AFPs are mainly small, cysteine-rich, cationic secretory compounds.

**NFAP2** (IATSPYYA**C**N**C**PNN**C**KHKKGSG**C**KYHSGPSDKSKVISGK**C**EWQGGQLN**C**IAT; 52 aa; 5.6 kDa; pI 8.7), secreted by *Neosartorya (Aspergillus) fischeri*, inhibits the growth of *Candida* species (MIC for *C. albicans* 1.1 μM) and its fungal cell-killing activity is linked to pore formation in the membrane. In an in vivo murine model of vulvovaginitis, NFAP2 significantly reduced the burden of fluconazole-resistant *C. albicans*, and its co-administration with fluconazole further enhanced the therapeutic efficacy [226].

**PAF** (AKYTGK**C**TKSKNE**C**KYKNDAGKDTFIK**C**PKFDNKK**C**TKDNNK**C**TVDTY-NNAVD**C**D; 55 aa; 6.25 kDa; pI 8.9), secreted by *Penicillium chrysogenum*, contains 13 lysines and 3 disulfide bridges resulting in a compact and highly stable β-barrel fold [227,228]. PAF inhibits the growth of *A. fumigatus* and *C. albicans*. Its mechanism involves the hyperpolarization of the fungal membrane, an increase in intracellular ROS and induction of an apoptosis-like cell death, with active internalization of the peptide into the cytoplasm [229]. PAF shows low toxicity to human cells and efficacy in a mouse model of invasive pulmonary aspergillosis, making it a candidate lead against azole-resistant *Aspergillus* infections.

**PeAfpA** (VLYTGQ**C**FKKDNI**C**KYKVNGKQNIAK**C**PSAANKR**C**EKDKNK**C**TFDSYDRKVT**C**DFRK; 57 aa; 6.64 kDa; pI 9.5), secreted by *Penicillium expansum*, forms five antiparallel β-strands folded into a compact β-barrel, stabilized by 3 disulfide bonds. It is active against filamentous fungi and yeasts at low micromolar concentrations (MIC for *C. albicans* 1.2 µM) [230]. Using the model yeast *Saccharomyces cerevisiae*, PeAfpA was found to be a multitarget antifungal agent acting on multiple pathways [231].

A small cationic peptide **SP1** (IRIAINGFGRIGRLVLRLALQRKDIEVVA; 29 aa; 3.3 kDa; pI 11.8) corresponds to the N-terminal fragment of *S. cerevisiae* glyceraldehyde-3-phosphate dehydrogenase (GAPDH), which is secreted during wine fermentation to outcompete other yeast competitors. Its activity against *C. albicans* and *A. fumigatus* is quite low (MICs ≥ 128 µM), while it acts effectively against *Cr. neoformans* (MICs 4–8 µM). The exposure of phosphatidylserine, DNA fragmentation, increased Ca^2+^ concentrations in the cytoplasm, mitochondrial dysfunction, and elevated ROS levels indicate that SP1 can induce apoptosis-like cell death [232].

### 4.6. Engineered Peptide Variants

A large number of synthetic short peptides have been generated based on the structures of natural AFPs from various classes. Most of these peptides are composed of 12–16 aa, enriched in cationic residues (Arg, Lys), which confer a net positive charge (+3–+6), and hydrophobic residues (Leu, Trp), responsible for high membrane affinity. Acetylation and amidation of the N- and C-terminal residues enhance the stability of peptides against proteolytic degradation. The substitution of L-amino acids by their D-enantiomers represents a well-established approach to enhance both the safety and efficacy of AFPs.

The synthetic peptide **RF3** (RIGRFLLFRGIRRIGRFL-NH_2_; 18 aa; 2.3 kDa; pI 14) was designed on the basis of the α-helical region of porcine myeloid AMP. This membranotropic AFP is characterized by moderate hydrophobicity, exhibits potent activity against fluconazole-resistant strains of *C. albicans* (MIC 4–8 μM) and modest hemolytic effects [233]. The lysine-enriched amphibian-derived temporin B analog, **TB_KKG6K** (KKLLPIVKNLLKSLL; 15 aa; 1.7 kDa; pI 11.2), exhibits potent fungicidal activity against *C. albicans* (MIC_90_ and MFC 2 μM), effectively reduces fungal biofilm maturation on silicone elastomers and is characterized by low cytotoxicity. The synthetic peptide **GW4** (GRWRWWWRWR-NH_2_; 10 aa; 1.6 kDa; pI 14) with N-terminal glycine inhibits the growth of *C. albicans* (MIC 6.25 μM), exhibits the capability to inhibit and eliminate fungal biofilms and causes hemolysis of erythrocytes. Both TB_KKG6K and GW4 disrupt the cellular membrane and facilitate intracellular ROS accumulation [234,235]. **P19** (RRFSFWFSFRR-NH_2_; 11 aa; 1.6 kDa; pI 14) exhibits anticandidal activity (MIC for *C. albicans* strains 2–4 μM), eradicates biofilms, and disrupts the fungal cell membrane, likely via binding to multiple membrane lipids [236]. The synthetic polyarginine peptide **NP339** (RRRRRRRRRRRRR; 13 aa; 2 kDa; pI 13.2) targets the fungal cell membrane and exhibits fungicidal activity against pathogenic fungi (MIC 2 or 16 μM for *C. albicans* or *C. auris* and *A. fumigatus*, respectively). NP339 was not effective in the disseminated candidiasis model, but substantially decreased fungal burden in both the vaginal candidiasis and oropharyngeal candidiasis murine models [237]. **RP557** (RF**C**WKV**C**YKGI**C**FKK**C**K-NH_2_; 17 aa; 2.1 kDa; pI 10.3), a peptide rationally derived from the disulfide-rich β-sheet AMP tachyplesin I, exhibits potent activity against both susceptible and fluconazole-resistant *Candida* isolates (MICs against *C. albicans* 7.5–32 μM), as well as against *Cr. neoformans* (MICs 2–4 µM), causing fungal damage via disruption of membrane integrity, reducing preformed biofilms, and inhibiting de novo biofilm formation. Notably, the peptide demonstrates substantial in vivo efficacy in a rat model of vulvovaginal candidiasis [238,239]. Another tachyplesin I analog **TP11A** (KW**C**FRV**C**YRGA**C**YRR**C**R-NH_2_; 17 aa; 2.2 kDa; pI 10.6) is less effective *against C. albicans*, but has a 64-fold higher activity/toxicity index than tachyplesin I [181]. **PL-18** (or HPRP-A2, or Gosteganan) (Ac-FKKLKKLFSKLWNWK-NH2; 15 aa; 2.04 kDa) is an all-D-amino-acid, N-terminally acetylated and C-terminally amidated α-helical membranotropic peptide derived from the N-terminal region of *Helicobacter pylori* ribosomal protein L1. This peptide exhibits antibacterial and antifungal activity (MIC 2 or 8 µM for *C. albicans* and *A. fumigatus*, respectively), inhibits *C. albicans* biofilm formation and disrupts biofilm structure. PL-18 acts synergistically with chlorhexidine acetate, and this combination demonstrates efficacy in the mouse and rat vaginitis models caused by bacteria or *C. albicans* [240,241]. PL-18 is currently being evaluated in a randomized, double-blind, placebo-controlled phase Ib/II clinical trial (CTR20232467) for the treatment of uncomplicated vulvovaginal candidiasis [242]. **CZEN-002** ([Ac-**C**KPV]_2_; 8 aa; 0.97 kDa) is a non-membranolytic synthetic disulfide-linked dimer derived from the C-terminal Lys-Pro-Val (KPV; α-MSH_11–13_) sequence of human α-melanocyte-stimulating hormone (α-MSH). CZEN-002 possesses immunomodulatory and anti-inflammatory effects, exhibits activity against susceptible and azole-resistant *Candida* spp. including *C. albicans* (effective at a concentration of 1 µM) and reduces the fungal burden in a rat model of *C. albicans* vaginitis [243,244]. CZEN-002 demonstrated both high efficacy and a favorable safety profile following topical administration in patients with vulvovaginal candidiasis during phase I/IIa clinical trials [245].

### 4.7. Translational Barriers to the Clinical Application of AFPs

**Figure 3:**
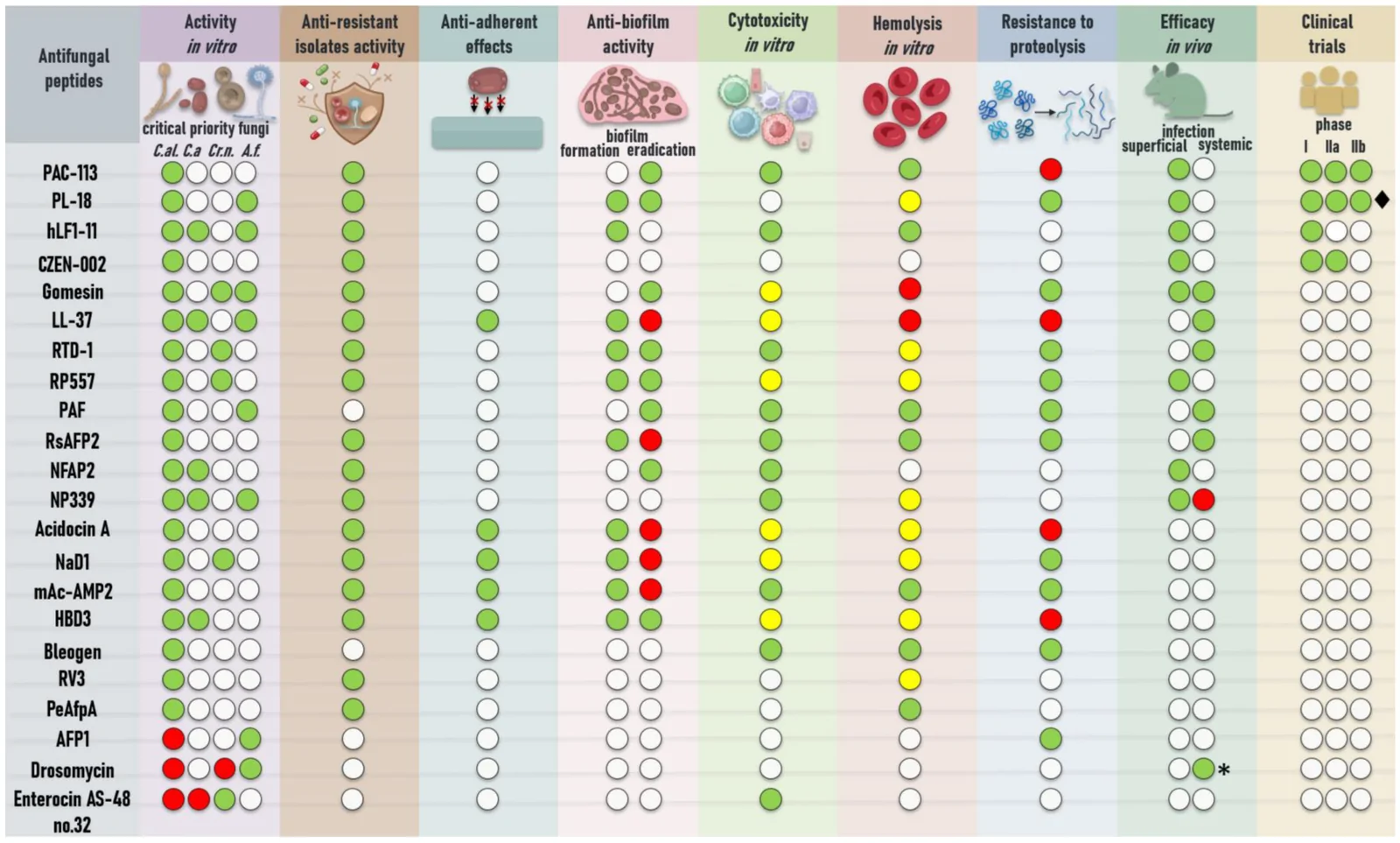
Translational evidence ladder for representative AFPs active against WHO critical priority fungi (additional references used for the figure are [246,247,248,249,250,251,252,253,254,255,256,257,258,259,260,261,262,263,264,265,266,267,268,269,270,271,272,273,274,275,276,277,278,279,280,281,282,283,284]). The green or red color of the circles indicates the presence or absence of efficacy of AFPs in the cases of: planktonic fungal cells of *C. albicans* (*C.al.*), *C. auris* (*C.a.*), *Cr. neoformans* (*Cr.n.*) and *A. fumigatus* (*A.f.*); resistant fungal isolates (predominantly azole-resistant *C. albicans* strains); adhesion of fungal cells to plastic or epithelial cells; biofilm formation or eradication; superficial (vulvovaginal or oropharyngeal candidiasis, gingivitis) or systemic (disseminated candidiasis or aspergillosis) fungal infections in animal models (mice, rats or dogs); clinical trials. The green, yellow or red color of the circles indicates low (hemolysis is absent, 50% cytotoxic concentration (CC_50_) > 100 μM), moderate (hemolysis at AFP concentrations above the MIC, CC_50_ 10–100 μM), or high toxicity (hemolysis at MIC, CC_50_ < 10 μM) of AFPs towards erythrocytes or other human cells in vitro. The green or red color of the circles also indicates resistance or, conversely, susceptibility of AFPs to proteolysis by various proteases, including serum, gastrointestinal or *C. albicans* enzymes. The white color of the circles shows the absence of data in all cases. *—Drosomycin expression in *imd*; *spz* double-mutant *Drosophila* (that do not express any of the known AMP genes) increased resistance to *A. fumigatus*. ♦—PL-18 has been included in a phase II clinical trial in China (IIa or IIb—not specified).

The color-coded matrix presented in Figure 3 provides a systematic overview of the translational evidence for a representative sample of the AFPs described in this review. Several key patterns emerge from this analysis.

The vast majority of AFPs have been evaluated against *C. albicans*, whereas data for other WHO critical priority fungi, particularly the emerging multidrug-resistant pathogen *C. auris*, remain scarce, highlighting a priority area for future research. A limited number of peptides, including hLF1-11, NP339, gomesin, and LL-37, exhibit broad-spectrum fungicidal activity, while others, such as AFP1, drosomycin, and the enterocin AS-48 fragment no. 32, are active against only a single species. While a narrow spectrum undoubtedly limits the potential clinical applications of AFPs, it is worth noting that some recently approved antifungals such as oteseconazole and ibrexafungerp are recommended only for specific indications, particularly recurrent vulvovaginal candidiasis.

Notably, virtually all the AFPs tested are active against resistant fungal strains. However, the available data are heavily skewed toward azole-resistant *C. albicans*. Future investigations that include other critical priority fungal species resistant to azoles as well as to other classes of conventional antimycotics would yield a more comprehensive assessment. Many AFPs show activity against *C. albicans* biofilms, yet this activity more commonly involves inhibition of biofilm formation than eradication of mature biofilms. However, a subset of AFPs, including PL-18, RTD-1, RP557, and HBD3, shows promise for treating biofilm-associated infections.

A significant limitation, particularly for the systemic application of AFPs, is their potential toxicity to mammalian cells in vitro, although the severity of these effects depends on their mechanism of action. Cationic, amphipathic AFPs with membranolytic action can damage both fungal and host cell membranes as seen with LL-37, gomesin, acidocin A, and the PI(4,5)P_2_-targeting plant defensin NaD1. It is worth noting, however, that in contrast to LL-37, gomesin did not exhibit toxic effects in animal model experiments. Nevertheless, analogs of gomesin (DsGom), NaD1 (NaD1-2-4) and tachyplesin I (TP11A) possess antifungal activity while exhibiting a much more favorable in vitro toxicity profile. At the same time, AFPs with fungal-specific targets (for example, chitin-binding hevein-like mAc-AMP2 or GlCers-binding plant defensin RsAFP2) or those with complex mechanisms affecting both the fungal membrane and intracellular targets (such as PAC-113, hLF1-11, PAF) show low or no toxicity. These findings indicate that a favorable therapeutic window can be achieved through optimization of AFP structure (sequence, charge, and hydrophobicity).

A further limitation hampering the clinical application of AFPs is their rapid degradation by host and fungal proteases, which would significantly reduce their half-life in human fluids. However, this property is intrinsic to the peptide structure. Susceptibility to proteolysis has been documented for the α-helical peptides LL-37 and PAC-113, as well as for acidocin A, whose three-dimensional structure remains unknown. By contrast, AFPs with a compact, disulfide-stabilized fold, including natural peptides such as gomesin, RTD-1, NaD1, RsAFP2, PAF, mAc-AMP2, AFP1, and bleogen, as well as semi-synthetic analogs like PL-18 and RP557, are generally resistant to proteolytic degradation. An exception is HBD3, which, despite its disulfide bonds, exhibits variable protease sensitivity owing to its considerable conformational flexibility. Stability can be further improved by terminal modifications including N-terminal acetylation (PL-18), C-terminal amidation (RP557), or the presence of N-terminal pyroglutamate (gomesin, bleogen) as well as by substitution of L-amino acids with their D-enantiomers (PL-18). Furthermore, the therapeutic effect of AFPs may stem partly from their immunomodulatory activity and the bioactivity of their partial proteolysis products.

Animal model studies for infections caused by critical priority fungi have been conducted for some AFPs, with the vast majority focusing on *C. albicans* and only rarely on *A. fumigatus*, whereas data for *C. auris* and *Cr. neoformans* are completely absent. Preclinical in vivo efficacy has been demonstrated primarily in superficial infection models, particularly vulvovaginal candidiasis, for natural AFPs (gomesin, RsAFP2, NFAP2) and engineered peptide variants (PL-18, CZEN-002, PAC-113, hLF1-11, RP557, NP339). In addition, five natural AFPs have shown efficacy in systemic infection models, including disseminated candidiasis (RsAFP2, RTD-1, gomesin) and pulmonary aspergillosis (LL-37, PAF). To enable successful clinical translation of AFPs, preclinical in vivo testing must be expanded to cover all four critical pathogens, as well as models that recapitulate biofilm-associated and chronic infections.

Analysis of the available data reveals a substantial gap between the preclinical findings for AFPs and their clinical translation. Among the numerous AFPs described in this review, only four have progressed to clinical trials, and none have advanced beyond phase II. In a phase II study of PAC-113 for oral candidiasis (NCT00659971), the peptide demonstrated safety and efficacy comparable to those of nystatin. However, the developer, Pacgen Biopharmaceuticals, apparently chose to redirect its application toward over-the-counter (OTC) and dental-care products. In phase I/IIa clinical trials, CZEN-002 demonstrated an excellent safety profile, good tolerability, and high efficacy upon topical administration in patients with vulvovaginal candidiasis, including activity against azole-resistant *C. albicans*. However, the planned phase IIb study was placed on hold by the developer, Zengen, Inc. Notably, hLF1-11, despite successful phase I safety data for systemic administration, was withdrawn before enrollment in a phase IIa trial for candidemia (NCT00509834) due to AM-Pharma’s decision. PL-18 (Gosteganan) is undergoing phase Ib/II trials for the treatment of uncomplicated vulvovaginal candidiasis in China (CTR20232467), and its future prospects remain unknown.

In summary, AFPs exhibit substantial potential against WHO critical priority fungi, which justifies continued research efforts. Their transition to clinical practice, however, is likely to be impeded not only by intrinsic biological shortcomings of the peptides, but also by economic and regulatory challenges confronting biotech developers.

## 5. Resistance to Antifungal Peptides and Its Possible Mechanisms

In the clinical microbiology laboratory, resistant strains have MICs above the clinical breakpoints as established by standardization organizations, e.g., CLSI and EUCAST. However, in research laboratories, in vitro resistance refers to a strain that is less susceptible and has a higher MIC for the tested compound than a control or reference strain. Studies on how rapidly critical priority fungi, mainly *Candida* species, adapt to AFPs during prolonged in vitro exposure are typically performed using serial passages at sub-inhibitory AFP concentrations or by in vitro experimental microevolution with stepwise increases in peptide concentration.

Available studies, although limited in the number of strains, duration of exposure, and experimental design, frequently report either failure to select an increase in MIC or smaller and slower MIC changes for several AFPs than for conventional antifungals of different classes used as comparators, including fluconazole, AmB, and caspofungin (Table 3). These observations indicate a low propensity for resistance selection for many AFPs under the tested conditions. The most likely explanation is that the mechanism of action of such molecules often does not involve a single target, but rather includes membranotropic effects, damage to the cell envelope, and other parallel processes, making the selection of classical stable resistance more difficult. It is worth noting, however, that only a limited number of studies have performed subsequent characterization of the resultant resistant fungal strains. Strains displaying reduced susceptibility to AFPs are rarely subjected to further experiments assessing the stability of the resistant phenotype after peptide withdrawal, population heterogeneity, fitness costs, mutational changes, and other molecular mechanisms (Table 3).

Across several repeated-exposure studies, AFP treatment resulted in either no detectable MIC increase over the reported passage series or only modest shifts in susceptibility, while conventional antimycotics often produced larger MIC changes under the respective experimental conditions. For example, for the synthetic peptide RF3, which has a dual-target antifungal mechanism of action, the MIC remained unchanged, while fluconazole showed a greater increase in MIC [233]. Similarly, microevolution experiments with TB_KKG6K showed only limited changes in *C. albicans* susceptibility, whereas serial exposure to GW4 produced no detectable MIC increase; substantially greater changes in susceptibility were observed with fluconazole [234,235]. This is consistent with the membranotropic mechanism of action of these peptides, which includes binding to lipid targets, membrane depolarization, increased membrane permeability, and intracellular ROS production [285,286]. Likewise, serial passaging with NP339 and RP557 did not increase MIC values, in contrast to conventional antifungals of different classes [237,238]. Repeated exposure to P19 and NpRS (RSLNLLMFR; 9 aa; 1.2 kDa; pI 12.1) from *Allium sativum* L. bulbs resulted in only slight increases in MICs, and the stability of these changes after peptide withdrawal was not assessed. The limited MIC changes observed for these peptides may be associated with the membrane-disruptive and fungicidal activity of P19 [236], whereas for NpRS, they may result from a combination of membrane damage, interference with intracellular processes, including ribosome-related pathways, and downregulation of *CDR1* expression [287]. For both susceptible and resistant *C. albicans* strains ATCC 18804 and ATCC 10231, exposure to the membrane-active LL-37 resulted in only a slight MIC increase after 24 serial passages, whereas no detectable MIC change was observed for the tobacco defensin NaD1 over the same passage period [174]. Serial passaging with the chitin-binding hevein-like peptide mAc-AMP2 diminished fungal susceptibility, but caspofungin, which also affects the fungal cell wall, caused a more pronounced increase in the MIC [174]. Transient adaptation likely occurred in this case, yet the effect was reversed when the peptide was withdrawn; however, its molecular mechanisms were not explored. Although most of these studies have been conducted using *Candida*, similar observations are beginning to emerge for other fungi as well. In particular, for *A. fumigatus*, no MEC (determined as the lowest peptide concentration inducing morphological changes) increase was observed for ETD151, a mutant analog of insect defensin ARD1, which also targets the fungal membrane, possibly via GlcCer binding [194].

Given the rarity of documented cases of fungal resistance to AFPs, the potential mechanisms underlying such resistance remain largely unexplored. The mechanism of resistance development has been studied for the plant defensin NaD1, but *S. cerevisiae* was used in these experiments. It was shown that resistance to NaD1 develops more slowly than resistance to caspofungin and is polygenic in nature, since no single knockout reproduced the level of resistance reached in the evolved lines. The NaD1-resistant strains showed enhanced growth under hyperosmotic stress, together with increased sensitivity to cell wall stressors. Cross-resistance to some plant defensins was observed: these strains showed reduced susceptibility to DmAMP1 and HXP4, but not to NaD2, suggesting partially overlapping but not universal protective mechanisms [288]. The NFAP2-adapted strains exhibited no morphological alterations and harbored non-silent mutations in only two genes. This phenotype was associated with decreased tolerance to cell wall stress, heat, and UV exposure [289]. Repeated exposure to histatin 3 resulted in an approximately 5-fold reduction in *C. albicans* susceptibility [291]. Notably, two primary derivatives lost this reduced susceptibility after long-term storage, indicating that the initially selected phenotype was not stable. Subsequent analysis showed that the resistant phenotype was accompanied by impaired metabolic function, reduced oxygen consumption, and alterations in multiple intracellular pathways, indicating that this is again not a simple case of mutation in a single target but rather a more complex cellular reprogramming [292].

Along with the above-mentioned experimental approaches that induce fungal resistance in vitro, additional mechanistic information can be obtained from mutant or knockout fungal strains, and from strains with altered gene expression. Such studies do not demonstrate the evolution of resistance per se, partly because mutant or knockout fungal strains may undergo significant compensatory changes in addition to being significantly less fit for survival in nature, but they can identify cellular pathways and molecular determinants that may contribute to reduced AFP susceptibility. Collectively, these studies indicate that decreased intracellular accumulation of the AFPs, remodeling of the fungal cell wall and membrane, and activation of regulatory stress-response pathways may contribute to reduced fungal susceptibility to AFPs, as detailed below.

The antifungal efficacy of AFPs is often dependent on their intracellular accumulation, which is governed by the relative rates of transporter-mediated cellular uptake and pump-mediated efflux. For example, knockout studies in *C. albicans* and *C. glabrata* showed that histatin 5’s fungicidal activity depends on Dur3- and Dur31-mediated cellular uptake [293]. Conversely, overexpression of the multidrug efflux pump MDR1 and other Mrr1 target genes confers reduced susceptibility to this AFP [294]. In a screen of the *S. cerevisiae* deletion library, genes associated with the transport of polyamines and other positively charged molecules were identified as regulators of NaD1 activity [295,296].

As noted above, certain AFPs target fungal-specific membrane lipids, including M(IP)_2_C (plant defensin DmAMP1) and GlcCer (plant and insect defensins RsAFP2 and heliomicin). These AFPs lack cytotoxic properties, but mutant fungal strains deficient in these specific lipids show reduced susceptibility to them [193,296,297,298]. Loss of negatively charged N-linked phosphomannans from the cell wall of *C. albicans* reduces susceptibility to dermaseptin S3 (1–16) [299], whereas the surface proteins Ssa1/2 are required for the anticandidal activity of HBD2 and HBD3 [300].

Changes in the energetic state of fungal cells may also contribute significantly to reduced susceptibility to AFPs. Suppressed mitochondrial ATP synthesis renders the mutant *C. albicans* significantly less sensitive to histatin 5, which is accompanied by reduced intracellular accumulation of the peptide [301]. Screening of the *S. cerevisiae* deletion library indicated a role for mitochondrial functions (HsAFP1, NaD1, and NbD6), vacuolar transport (NbD6 and SBI6), and ATP transport (NaD1, NbD6, SBI6, and DmAMP1) in fungal susceptibility to plant defensins [296,302].

A number of studies show that different signaling pathways regulating the cellular response to external stress are also involved in fungal susceptibility to AFPs, in particular to plant defensins DmAMP1 [296] and HsAFP1 [302]. The calcium/calcineurin signaling pathway contributes to the cellular response of *C. albicans* to the MUC7 12-mer derived from salivary mucin MUC7 [303], whereas the cell wall integrity signaling pathway contributes to protection of *A. nidulans* against PAF from *Penicillium chrysogenum* [304]. Regulatory proteins Ssd1 and Bcr1 modulate the susceptibility of *C. albicans* to such human AFPs as LL-37 and HBD2 [305,306]. Genomic screening of a collection of *S. cerevisiae* deletion mutants revealed that genes involved in ribosomal functions, protein glycosylation, vacuolar and vesicular transport, as well as the induction of the RIM101 signaling pathway, including several components of the ESCRT sorting machinery, are involved in fungal susceptibility to different human salivary AFPs [307].

Thus, experimental data on resistance induction indicate that many AFPs, particularly those that target the fungal cell membrane and have multiple mechanisms of action, likely present a high barrier to fungal resistance development in vitro. This is predominantly attributed to their substantial molecular size relative to conventional antifungals, which allows AFPs to simultaneously engage multiple fungal targets, thereby complicating evasion through point mutations. From an evolutionary perspective, host organisms expand this multifunctionality by generating synergistic peptide repertoires through gene duplication and divergence, whereby a large proportion of eukaryotic AMPs are encoded by genomic clusters of paralogous genes. Moreover, structural modification of ribosomally synthesized peptides appears to be simpler than generating structural diversity among antifungal secondary metabolites, since the latter entails altering complex multi-enzyme pathways. It should be noted, however, that data regarding fungal resistance to AFPs are limited, as they were obtained from in vitro experiments rather than from in vivo clinical settings and primarily involved *C. albicans*, not other WHO critical priority fungal species.

## 6. Conclusions

Taken together, the presented data reveal that resistance to all major classes of antifungals (azoles, polyenes, echinocandins, and flucytosine) is emerging across the four WHO critical priority fungal pathogens: *C. albicans*, *C. auris*, *A. fumigatus*, and *Cr. neoformans*. This restricts the available therapeutic arsenal and highlights a pressing need for alternative antifungal strategies. With the exception of 5-FC, which is not used as monotherapy, resistance to azoles constitutes the most prevalent form of resistance among all four critical priority fungi. Among these pathogens, *C. auris* is of the greatest concern, as high population-level rates of fluconazole resistance, combined with emerging reduced susceptibility to other antifungal classes, frequently result in a multidrug-resistant profile. All four WHO critical priority fungal species employ a shared set of evolutionarily conserved molecular resistance strategies, including target modification and overexpression, activation of efflux systems, membrane lipid remodeling, and induction of stress-response pathways. However, species-specific differences including, in particular, high genomic plasticity of *C. auris*, cross-resistance to agricultural azoles in *A. fumigatus*, and the intrinsic structural resistance to echinocandins in *Cr. neoformans* determine unique resistance profiles of each pathogen and underscore the need for differentiated monitoring and therapeutic strategies.

The fact that resistance is developed not only to conventional antimycotics but also to novel antifungal agents currently being introduced into clinical practice, including those targeting previously unexploited molecular sites, is of particular concern. The most significant risks involve cross-resistance between fosmanogepix and azoles via efflux pump activation, between rezafungin or ibrexafungerp and echinocandins driven by target gene mutations, and between olorofim and the agricultural fungicide ipflufenoquin, which share a common molecular target.

Current approaches to circumventing antifungal resistance encompass combination therapy, drug repurposing, targeted delivery, antibiofilm strategies, immunotherapy, and the application of naturally occurring antifungal agents. While some of these strategies have shown promise in vitro, further preclinical and clinical studies are required to validate their therapeutic potential.

AFPs derived from diverse sources, including plants, animals, humans, bacteria, and fungi, as well as their synthetic analogs, are of particular interest. They are active against WHO critical priority fungi, encompassing resistant clinical isolates, exhibit anti-adherent and antibiofilm properties, prevent fungal invasion, and demonstrate efficacy in animal models of superficial and systemic fungal infections. These peptides are characterized by remarkable structural diversity and act on molecular targets that fundamentally differ from those of conventional antimycotics. Their modes of action comprise cell wall disruption, interaction with universal or fungal-specific lipids, membrane permeabilization, and intracellular effects, including accumulation of ROS, mitochondrial dysfunction, and induction of apoptosis. In vitro data indicate that many AFPs exhibit slow or no development of resistance in critical priority fungi during prolonged exposure, compared with conventional antifungals of different classes, including fluconazole, AmB, and caspofungin. In rare cases, when increases in MICs are observed, they involve polygenic metabolic alterations or polygenic adaptation rather than single-target mutations, or are reversed when the peptide is withdrawn. Thus, the multifaceted mechanism of action of most AFPs, encompassing effects on the fungal cell wall, plasma membrane, and intracellular targets, may impose a higher barrier to resistance development among clinically important fungi at least in vitro.

Despite promising in vivo data for several AFPs, their clinical application is hampered by proteolytic instability, potential toxicity and immunogenicity, and the challenges and high costs of large-scale manufacturing. These limitations might be partially mitigated by chemical modifications of the peptides, such as amidation, cyclization, PEGylation, and the incorporation of non-natural amino acid residues as well as by the use of suitable delivery systems [308]. Integration of artificial intelligence and machine learning into AFP discovery and rational design, peptide bioengineering, and nanodelivery strategies might also help to address these challenges [309,310,311]. Overall, antifungal peptides represent a promising platform for the development of next-generation antimycotics, potentially helping to overcome resistance in WHO critical priority fungal pathogens, although further in vivo validation is needed.

## Figures and Tables

**Table 1:** Conventional antifungal drugs and resistance mechanisms in WHO critical priority fungi.

Conventional Antifungals	Source	Mechanism of Antifungal Action	Species	Mechanism of Fungal Resistance
**Azoles** (fluconazole, voriconazole, posaconazole, itraconazole, isavuconazole)	Synthetic	Azoles selectively inhibit lanosterol 14-α-demethylase (ERG11/CYP51), a cytochrome P450–dependent enzyme that catalyzes a rate-limiting step in the biosynthesis of ergosterol from lanosterol. This leads to the accumulation of 14-methylsterols in the plasma membrane, which disrupts the integrity and fluidity of the lipid bilayer, impairs membrane permeability, and compromises the function of key membrane proteins and transport systems. Moreover, azoles promote the production of reactive oxygen species (ROS) and subsequent oxidative damage, potentiating cell death.	*Cr. neoformans*	*ERG11* amino acid substitutions; overexpression of ABC efflux pumps; *MSH2* mismatch repair defects [42,43].
			*C. albicans*	*ERG11* amino acid substitutions; *ERG11* overexpression; Upc2-mediated activation of sterol biosynthesis genes; overexpression of ABC and MFS efflux pumps [44,45].
			*C. auris*	*ERG11* amino acid substitutions and overexpression; *TAC1b* gain-of-function and efflux pump overexpression; subtelomeric deletions and SNP accumulation [44,46].
			*A. fumigatus*	*cyp51A* alterations (promoter tandem repeats and/or point mutations); overexpression of efflux pumps; *HapE* mutations; mitochondrial complex I alterations; cytochrome b5–CybE redox changes; *SrbA/AtrR* transcription factor dysregulation [47,48,49].
**Polyenes** (amphotericin B (AmB), nystatin, natamycin, candicidin, trichomycin, mepartricin)	Natural (*Strepto-myces* spp.)	Polyenes bind with high affinity to ergosterol in the fungal cell membrane (and to cholesterol in human cells with lower efficiency), leading to the formation of transmembrane pores. These pores facilitate the leakage of intracellular contents and disrupt osmotic and ionic balance, causing rapid fungal cell death. AmB can also assemble into extramembranous aggregates that sequester ergosterol from the lipid bilayer (“sterol sponge” mechanism) and induce ROS-mediated oxidative damage.	*Cr. neoformans*	Defects or alterations in sterol isomerase (Erg2) and related sterol pathway enzymes (Erg3, Erg6); dysregulation of transcription factors (Sre1, Mbs1, Hob1) controlling ergosterol biosynthesis [42,50].
			*C. albicans*	*ERG*-pathway mutations (*ERG3*/other *ERG* genes); Upc2p-mediated dysregulation of sterol biosynthesis; Hsp90-dependent stress response pathways [44,50].
			*C. auris*	*ERG6* frameshift mutation abolishing ergosterol production. *ERG3* frameshift mutation leading to altered sterol composition [46,50].
			*A. fumigatus*	Incompletely understood; data do not consistently map to classical ergosterol biosynthesis genes [41,48,49].
**Echinocandins** (caspofungin, micafungin, anidulafungin)	Semi-synthetic	By noncompetitively inhibiting the β-(1,3)-D-glucan synthase complex, echinocandins disrupt β-(1,3)-D-glucan synthesis, compromise cell wall integrity, induce osmotic instability, and trigger cell lysis. Caspofungin promotes ROS accumulation and ROS-mediated apoptotic responses.	*Cr. neoformans*	Data are lacking as they are not used to treat cryptococcal infections [51,52].
			*C. albicans*	*FKS1* amino acid substitutions (e.g., S645F/P/Y, F641S) [53,54].
			*C. auris*	*FKS1* amino acid substitutions (e.g., S639F/P/Y, F635C) [46,53].
			*A. fumigatus*	*FKS1* amino acid substitution (e.g., S678P) [55].
**Nucleoside analogs** (5-flucytosine (5-FC))	Synthetic	5-FC enters fungal cells through a cytosine permease and is deaminated by cytosine deaminase to 5-fluorouracil (5-FU), which is metabolized further. 5-FU is converted to 5-fluorodeoxyuridylate (5-FdUMP), thereby inhibiting thymidylate synthase and downstream DNA synthesis, and to 5-fluorouridine monophosphate (5-FUMP), which is phosphoryla-ted to 5-fluorouridine triphosphate (5-FUTP) and incorporated into fungal RNA, causing its miscoding and impaired protein synthesis.	*Cr. neoformans* *C. albicans*	Loss-of-function mutations in pyrimidine salvage pathway genes: *FCY2*/*FCY21*/*FCY22* (cytosine permeases); *FCA1*/*FCY1* (cytosine deaminase); *FUR1* (uracil phosphoribosyltransferase) [39,56].
			*C. auris*	Loss-of-function mutations in pyrimidine salvage pathway genes *FCY1*, *FCY2*, *FUR1* [46,56].
			*A. fumigatus*	pH-dependent downregulation or dysfunction of FcyB [57].

**Table 2:** Translational status of resistance-overcoming strategies against WHO critical priority fungi.

Strategy	Representative Agents/ Approaches	In Vitro Activity	In Vivo Efficacy	Clinical Trials	Current Clinical Use
**Combination therapy**	ISZ + MIF; 5-FC + ECH or 5-FC + AmB; AmB + FLC	ISZ + MIF—multidrug-resistant *C. auris*; 5-FC + ECH and 5-FC + AmB—echinocandin- and polyene-resistant *C. auris*	ISZ + MIF therapy—in rabbit invasive aspergillosis model	ACTA trial: AmB deoxycholate + 5-FC/FLC as induction therapy for HIV-associated cryptococcal meningitis; AMBITION-cm trial: single high-dose liposomal AmB + 5-FC + FLC	AmB + 5-FC for induction therapy of cryptococcal meningitis, central nervous system infections, and *Candida* endocarditis (AmB + FLC when 5-FC is unavailable)
**Drug** **repurposing**	Colistin, erythromycin, tamoxifen, statins, ibuprofen, neuroleptics	Colistin—multidrug-resistant *Candida* and *Cryptococcus*; erythromycin invigorates AmB against *Candida*, and *Cr. neoformans*; tamoxifen—*C. albicans* and *Cr. neoformans*; statins—*Candida*, *Cr. neoformans* and *A. fumigatus*; ibuprofen—*Cryptococcus*; neuroleptics—all WHO critical priority fungi	—	—	—
**Targeted** **delivery**	Liposomal AmB or NYS; DC-SIGN-functionalized AmB liposomes; NYS-loaded niosomes	Liposomal AmB or NYS, DC-SIGN-functionalized AmB liposomes, NYS-loaded niosomes—*Candida*, *Cryptococcus*, *Aspergillus*	DC-SIGN-AmB liposomes and liposomal NYS—in murine models of invasive candidiasis and aspergillosis; NYS-loaded niosomes—invasive candidiasis	Liposomal NYS (Nyotran^®^) underwent clinical trials for the treatment of different systemic infections but was not approved by the FDA	Liposomal AmB (AmBisome^®^, Fungisome^®^) for different invasive fungal infections when the use of triazoles or ECHs is undermined
**Antibiofilm strategies**	Combination of conventional antimycotics with antiseptics, calcineurin inhibitors, statins, antibiotics, natural compounds, or other antifungal agents	Calcineurin inhibitors (FK506, cyclosporine A) synergize with FLC against *C. albicans* biofilms, restoring fungicidal activity	Calcineurin inhibitor + FLC—in rat central-venous-catheter biofilm model	—	—
**Immuno-therapy**	Vaccines, cell-based therapy, cytokine-based therapy, antibody-based therapy	SRCD5CAR-based CAR-NK cells active against *C. albicans* and *Cr. neoformans*	Vaccines in a mouse model: VesiVax^®^ Af3/9 and ΔsglA *A. fumigatus* conidia vaccine—invasive aspergillosis; Cda2-Pep1 GP and CDA1-LNP mRNA vaccine—cryptococcosis; heat-killed fbp1Δ mutant *Cr. neoformans* vaccine—broad-spectrum of invasive fungal infections. Cell-based therapy in mouse model: adoptive NK-cell transfer—aspergillosis; *A. fumigatus*-specific T-cell transfusion—aspergillosis and lethal infection with *C. albicans*; SRCD5CAR CAR-NK cells—invasive infections caused by *C. albicans*, *A. fumigatus*, and *Cr. neoformans*. GM-CSF/G-CSF—in a mouse model of aspergillosis. Efungumab—in mouse model of disseminated candidiasis	NDV-3/NDV-3A (phase Ib/IIa); Candi5V (phase I/II); PEV7 (phase I); *A. fumigatus*-specific T cells (small clinical trials); IFN-γ (phase I, II as an adjuvant immunotherapy to combat critical-priority fungal infection)	Granulocyte transfusion for neutropenic patients with invasive mycoses
**Natural** antifungal compounds *	Fatty acids, plant terpenoids, saponins and flavonoids, fungal secondary metabolites	Broad activity against *Candida*, *A*. *fumigatus*, and *Cr. neoformans*; camphor, eucalyptol, and flavonoids also inhibit fungal biofilm formation	—	—	—

* Data on antifungal peptides are discussed in detail in the following sections. FLC—fluconazole; ISZ—isavuconazole; ECH—echinocandin; MIF—micafungin; NYS—nystatin.

**Table 3:** Induction of resistance by prolonged AFP exposure in serial passage experiments or in vitro microevolution experiments.

AFP	Source	Mechanism of Action	Fungal Species Tested	Resistance and Possible Mechanism	References
**RF3**	Synthetic rationally designed α-helical peptide	Plasma membrane permeabilization, intracellular ROS accumulation	*C. albicans*	No MIC increase after 10 serial passages; fluconazole showed 8 × MIC	[233]
**TB_KKG6K**	Amphibian-derived temporin B analog	Plasma membrane depolarization and permeabilization, intracellular ROS accumulation, destruction of subcellular structures in yeast cells	*C. albicans*	Adaptation to MIC_90_, but not to 2 × MIC_90_ in an in vitro microevolution experiment; fluconazole adaptation reached 32 × MIC_90_	[234,285]
**GW4**	Synthetic N-glycine-capped derivative of peptide W4	Plasma membrane permeabilization, intracellular ROS accumulation, mitochondrial membrane depolarization	*C. albicans*	Fluctuations in the MIC value, ranging from 1× to 2× during 20 serial passages; fluconazole showed 32 × MIC	[235,286]
**P19**	Synthetic central-symmetric short peptide	Lipid binding and membrane dysfunction	*C. albicans*	Fluctuations in the MIC value, ranging from 0.5× to 2× during 20 serial passages; fluconazole and AmB showed 1024 × MIC	[236]
**NpRS**	Garlic (*Allium sativum* L.)	Plasma membrane permeabilization, disruption of ribosome-related intracellular pathways, downregulation of the gene *CDR1*	*C. albicans*	Fluctuations in the MIC value, ranging from 1× to 2× during 21 days of serial passaging; fluconazole showed 16 × MIC after 8 serial passages	[287]
**RP557,** **RP554,** **RP556**	Synthetic peptides rationally derived from tachyplesin I	Plasma membrane permeabilization	*C. tropicalis*	No MIC increase after 9 serial passages	[238]
**NP339**	Synthetic polyarginine peptide	Plasma membrane permeabilization	*C. auris*, *C. albicans*, *C. glabrata*, *C. tropicalis*	No MIC increase after 30 serial passages; caspofungin, fluconazole, and AmB showed an increase in MICs ranging from 4× to 8×, 2× to 256× or 4×, respectively (depending on the strain)	[237]
**ETD151**	Engineered from a sequence shared by the insect-derived peptides, heliomicin and ARD1 isolated from *Heliothis virescens* and *Archaeoprepona demophon*	Lipid binding (GlCer) and membrane dysfunction, triggering fungal stress response and disruption of cell wall homeostasis	*A. fumigatus*	No minimal effective concentration (MEC) change after 16 serial passages	[194]
**NaD1**	Plant defensin from the flowers of *Nicotiana alata*	Interaction with fungal cell wall, lipid binding (PI(4,5)P_2_, PA), plasma membrane permeabilization, intracellular ROS accumulation	*S. cerevisiae*, *C. albicans*	In *S. cerevisiae*, the MIC increased 32-fold after 20 serial passages and it was associated with polygenic adaptation involving cell wall remodeling and osmoregulatory pathways; caspofungin showed 32 × MIC after 15 serial passages. No MIC increase was observed in *C. albicans* after 24 serial passages; caspofungin showed 8 × MIC after 21 serial passages	[174,288]
**mAc-AMP2**	Modified hevein-like peptide from *Amaranthus* *caudatus* seeds	Chitin-binding activity, membrane permeability disruption (at higher concentrations)	*C. albicans*	The MIC increased 4-fold during 24 serial passages, but returned to baseline after peptide withdrawal; caspofungin showed 8 × MIC after 21 serial passages	[174]
**LL-37**	Human cathelicidin	Disruption of cell wall integrity, plasma membrane permeabilization, modulation of signaling pathways, inhibition of cell cycle progression, intracellular ROS accumulation, disruption of endoplasmic reticulum homeostasis, and formation of autophagy-like structures	*C. albicans*	MIC increased 2-fold during 24 serial passages; caspofungin showed 8 × MIC after 21 serial passages	[174,199]
**NFAP2**	*Neosartorya (Aspergillus)* *fischeri*	Lipid binding and membrane dysfunction, reduces the metabolic activity by interacting with Gad1p, Atp1p, and Eno1p	*C. albicans*	Adaptation to the MIC, but not to 2 × MIC in in vitro microevolution experiment; fluconazole adaptation reached 32 × MIC. Tolerance is associated with mutations likely involved in stress responses and reduced AFP uptake	[289,290]
**Histatin 3**	Human salivary peptide	Binding to cell surface receptors, intracellular targeting, nonlytic ATP release	*C. albicans*	5-fold decrease in killing capacity after exposure to increasing concentrations; the phenotype was associated with impaired metabolism, reduced oxygen consumption, and multiple intracellular metabolic alterations	[291,292]

## Data Availability

The original contributions presented in the study are included in the article; further inquiries can be directed to the corresponding author.
